# Assessing Molecular Dynamics in Predicting Aptamer–Ligand Binding Thermodynamics: Insights from the OTA Binding Aptamers

**DOI:** 10.1021/acs.jcim.5c02643

**Published:** 2026-04-03

**Authors:** Alessio Olivieri, Federica Borzelli, Mauro Giustini, Marco D’Abramo

**Affiliations:** Department of Chemistry, Sapienza University of Rome, 00185 Rome, Italy

## Abstract

The targeting of nucleic acid platforms is of particular interest in biochemistry and pharmaceutical applications. Among nucleic-based structures, aptamers, short, synthetic oligonucleotides, stand out because of their tunable sequences, enabling highly selective recognition of molecules of different sizes. However, an accurate evaluation of aptamers’ affinity toward their targets remains elusive, as results obtained from different experimental techniques are often inconsistent. In this context, computational methods provide an appealing alternative for characterizing aptamer binding and structure. To this end, we selected two ochratoxin A binding aptamers as a case study to assess the ability of molecular dynamics simulations and alchemical free energy calculations to model the conformational dynamics and binding thermodynamics of these systems. Extensive classical molecular dynamics simulations were performed to characterize the aptamers’ structures in the absence of their ligand, which could not be determined experimentally due to the intrinsic flexibility of these sequences. Additional simulations on aptamer-ligand complexes provided atomistic details of the interactions underlying the corresponding aptamers’ preferential binding to their target compared with an analogue ligand that differs by a single atom. Lastly, alchemical free energy calculations were employed to estimate aptamers’ selectivity for the target over its analogue, expressed as relative binding free energy. Our estimates are in good agreement with experimental data. We expect these computational strategies to contribute to future protocols for aptamer design and evaluation, enabling a more rigorous assessment of their binding to biochemically relevant molecules.

## Graphical abstract

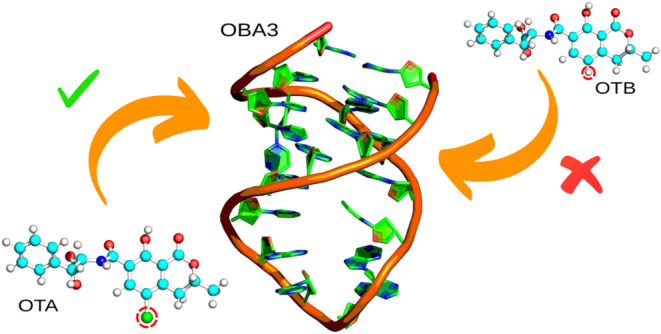

## Introduction

Aptamers are short, single-stranded oligonucleotides (either DNA or RNA) that can fold into well-defined three-dimensional structures, which confer them the ability to bind a wide array of molecular targets with high specificity and affinity. Aptamers are generated via SELEX (Systematic Evolution of Ligands by Exponential Enrichment), an *in vitro* selection process first introduced in 1990.^1–3^ Since then, aptamers have emerged as promising molecular recognition elements with broad applicability in diagnostics, therapeutics, biosensing, and environmental monitoring.^4–7^ Compared to traditional biorecognition molecules such as antibodies or enzymes, aptamers offer several advantages: high chemical and thermal stability, low immunogenicity, ease of chemical synthesis and modification, batch-to-batch reproducibility, and extended shelf life.^8,9^ Over the past three decades, different SELEX variants have been developed to improve the efficiency of aptamer selection, broaden the range of accessible targets, and reduce selection times.^10–12^ These innovations have allowed the selection of aptamers against an increasingly wide spectrum of targets, from small organic molecules to proteins, cells, and viruses. Nevertheless, aptamer technologies have not yet achieved widespread commercial success. One of the main obstacles to their wider application is the lack of standardized protocols for characterizing the affinity and specificity of aptamer-target interactions.^13–15^ Current validation methods, including equilibrium dialysis, fluorescence assays, ultrafiltration, and affinity chromatography using magnetic beads, are cost-effective and easy to implement though often yielding inconsistent results:^14^ estimates of binding constants for the same aptamer–target pair can differ by several orders of magnitude when obtained using different methods.^15^ Such discrepancies call into question the reliability and reproducibility of published data and undermine confidence in aptamer-based systems. Experimental methods such as surface plasmon resonance (SPR)^16^ and capillary electrophoresis (CE)^17^ provide higher accuracy and detailed kinetic information; however, they also introduce methodological constraints. SPR, for instance, requires immobilization of aptamers or targets on chips – often involving chemical linkers or surface modifications which may perturb the aptamer’s native conformation and alter its binding mechanism.^18^ CE, in turn, is poorly suited for evaluating interactions with low-molecular-weight ligands.^19^ These limitations become particularly problematic for small molecule targets (<1000 Da), which constitute a significant class of clinically and environmentally relevant chemicals.^19^ Furthermore, SELEX involves the multistep amplification of target-bound oligonucleotides through the polymerase chain reaction (PCR); as a result, sequences with superior amplification efficiency can be preferentially enriched even when their binding affinity is lower. In such cases, affinity can be improved through post-SELEX optimizations, employing strategies such as truncation, chemical modification, mutagenesis, and structure-guided approaches.^20–23^ To address these challenges, computational methods–particularly molecular dynamics (MD) simulations–are attracting a growing interest as tools for understanding and predicting aptamer-target interactions at an atomistic level.^24–30^ MD simulations can provide critical insights into aptamer folding pathways, conformational dynamics, and structural determinants of binding affinity and specificity. When used in conjunction with experimental data, MD can assist in the rational design of aptamers, helping to resolve ambiguous analysis results and even allowing prescreening aptamer candidates prior to empirical validation. These approaches are especially valuable for small molecule targets, where experimental techniques often struggle to capture weak or transient interactions.^25,28,30^ However, the integration of MD simulations into routine aptamer research remains limited, partly due to the computational expertise required. Building on this, we centered our case study on the ochratoxin A–binding aptamers. The OBAwt aptamer^31^ binds ochratoxin A (OTA) – a mycotoxin and a prevalent food contaminant – with high affinity and selectivity.^32,33^ The aptamer also recognizes ochratoxin B (OTB), a structural analogue of OTA lacking the chlorine atom in the isocoumarin ring (Figure 1), with a 100-fold lower affinity.^31^ More recently, two innovative sequences, namely, OBA3 and OBA33, were proposed on the basis of experimental structural information concerning OBAwt. These new aptamers exhibit enhanced selectivity and affinity toward OTA, while maintaining a negligible binding to OTB.^22,23^ In this study, we use *in silico* characterization to extend our investigation of the ligand-free aptamers OBA3 and OBA33, as well as their complexes with OTA and OTB, in solution. The aim of this work is 2-fold: to acquire more in-depth knowledge of the molecular mechanisms underlying the almost selective recognition of the two ochratoxins by the OBA3 and OBA33 aptamers, while evaluating the effectiveness of computational tools for aptamer development. Particular emphasis is devoted to comparing the thermodynamics of binding of OTA and OTB to the aptamer. Classical, unbiased molecular dynamics simulations revealed no binding or unbinding events, likely due to slow kinetics in both directions. A direct estimation of the free energy difference between binding and dissociation (closely related to the dissociation constant) thus requires enhanced sampling techniques such as steered molecular dynamics (SMD) and umbrella sampling (US). While these methods can, in principle, provide such estimates, their reliability is often constrained by the need to sample the system along a well-defined single reaction coordinate. This limitation can reduce both accuracy and reproducibility.^34–36^ An alternative to pathway-based methods is offered by alchemical free-energy calculations, which have evolved to be reliable tools for predicting relative binding affinities. These methods are frequently adopted as the final confirmatory step in the *in silico* drug-discovery process, serving to validate lead identification findings.^37,38^ Recent developments – most notably force-field recalibration procedures and bidirectional nonequilibrium approaches– have further improved the accuracy of relative binding free-energy estimations.^39,40^ On this basis, the nonequilibrium alchemical switching methodology is hereby adopted for the purpose of modeling OTA binding aptamer selectivity. This approach is especially well-suited for the case in question, where OTA and OTB differ by only a single atom (Figure 1).^41–45^

**Figure 1:**
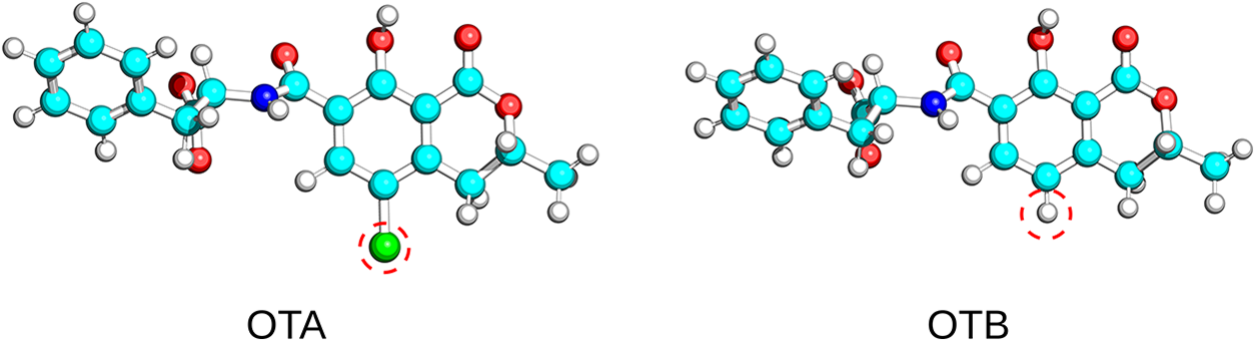
Ball-and-stick representation of ochratoxin A (OTA) and ochratoxin B (OTB). Circled atoms highlight the chlorine atom and the hydrogen atom in the isocoumarin ring of OTA and of OTB, respectively.

## Methods

### Molecular Dynamics Simulations

MD simulations were carried out using the GROMACS 2022.5 simulation package^46^ for the following six systems in water: the OBA3–OTA complex, the OBA3–OTB complex, the OBA3 aptamer in the absence of ligands, the OBA33–OTA complex, the OBA33–OTB complex, and the OBA33 aptamer in the absence of ligands. To allow direct comparison between the OTA and OTB complexes, identical protonation states were modeled: the carboxyl group was deprotonated and the phenolic group protonated, resulting in a net charge of −1 for both toxins. Additional simulations with phenolate OTA yielded the same results obtained when only the carboxyl group was deprotonated (data not shown). These findings are in agreement with experiments performed at different pHs.^22^

MD simulations were performed using the AMBER14SB force field with parmBSC1 corrections, optimized for nucleic acids.^47^ The topology and molecular parametrization of OTA and OTB were generated with the AnteChamber Python Parser interface (ACPYPE) program.^48^ To model the potential halogen bond between the chlorine atom of OTA and each of the aptamers, a positive site (dummy atom) was introduced along the extension of the C–Cl bond in the OBA3-OTA and OBA33-OTA systems to mimic the σ-hole. The parameters for this extra point of charge were adapted from previously reported values;^49^ among these, those derived for chlorobenzene, which most closely resembles the chemical environment of the chlorine atom in OTA and its halogen bonding behavior, were selected. The chlorine atom’s partial charge was adjusted to preserve the overall net charge of −1 for the molecule. This choice was supported by calculations of ESP charges for OTA (Table S1). The electronic structure (ESP) calculations were performed using the Gaussian 16 software package.^50^ To further validate the MD parameters for the OTA molecule, MM and QM energies were calculated; these proved to be in rather good agreement with the estimates for a similar system previously reported in the literature (Table S2).^49^ All simulations were conducted in the NVT ensemble. The molecular dynamics simulation protocol was implemented as follows. The initial geometries of both the aptamers and the mycotoxins (OTA and OTB) were taken from the NMR structures reported by Xu and collaborators^22,23^ (6J2W for OBA3 and7W9Nfor OBA33 in PDB), to enable comparison with available data. The two simulated systems, hereafter referred to as OBA3 (19 nucleobases) and OBA33 (33 nucleobases), are DNA-based oligonucleotides with sequences: 5′-d­(CGGGGCGAAGCGGGTCCCG)-3′ and 5′-d­(CGATCGGGTGTGGGTGGCGTAAAGGGAGCATCG)-3′, respectively. For the ligand-free systems, the structure reported for the aptamers in complex with OTA was used as starting coordinates for the simulations. For the OBA3 simulations, each system was placed in a cubic simulation box with edge length of approximately 6 nm. The boxes were then solvated with 6562, 6510, and 5512 TIP3P water molecules for OBA3-OTA, OBA3-OTB, and OBA3, respectively. The neutralization of the systems was achieved by adding Na^+^ ions as required. Energy minimization was initially performed by restraining the positions of the heavy ochratoxin atoms with a force constant of 500 kJ mol^–1^ nm^–2^. The system was then equilibrated at 250 K, using restraints for 1 ns. This was followed by heating at 300 K and equilibration for 1 ns with restraints and removing them for another 1 ns. Additional short equilibration steps, in which the box size was adjusted to reproduce the experimental water density, were performed as previously described in the literature.^51^ Similarly, each OBA33 system was placed in a cubic simulation box with edge length of approximately 7 nm and solvated with 11753, 11601, and 11611 TIP3P water molecules for OBA33-OTA, OBA33-OTB and OBA33, respectively. This was followed by neutralization, energy minimization, and pressure adjustment as described for the OBA3 systems. For the OBA33-OTA and OBA33-OTB systems, energy minimization was initially performed by restraining the positions of the ochratoxin heavy atoms with a force constant of 500 kJ mol^–1^ nm^–2^. The system was then equilibrated at 250 K, using restraints for 1 ns. This was followed by equilibration at 300 K for 1 ns under restraints and for 1 ns without restraints. Short equilibration steps in which the box size was adjusted to reproduce the experimental water density were performed. Finally, a longer unrestrained equilibration of 100 ns was performed at 300 K. For all simulations, long-distance electrostatic forces were determined using the Ewald Particle Mesh (PME) method^52,53^ with a cutoff radius of 1.1 nm. The same cutoff value was used for the van der Waals interactions. The temperature was held constant at 300 K using the velocity-rescaling thermostat with a stochastic term^54^ and a τ_T_ of 0.01 ps.

Each system was simulated for 1 μs; a time step of 2 fs was used for both production and equilibration. The LINCS algorithm was used to constrain bonds involving hydrogen atoms.^55^ To better capture the conformational flexibility of the ligand-free aptamers, five independent 1 μs simulations were performed under identical conditions for ligand-free OBA3 and ligand-free OBA33.

### Construction of Hybrid Topology

Alchemical free energy calculations require the construction of a nonphysical pathway that connects two thermodynamic states between which the free energy difference is evaluated. Progress along this path is driven by a coupling parameter, λ, which ranges from 0 to 1 over the entire process. In this study, λ = 0 corresponds to OTA, and λ = 1 corresponds to OTB. Intermediate alchemical states are modeled by creating hybrid topologies, mixtures of the two ochratoxins; as λ increases, the atom present only in OTA (the chlorine substituent) and its interactions are gradually removed, while the hydrogen present only in OTB is gradually introduced; this is implemented using dummy atoms (Figure 2). The pmx library^43^ was used to generate the necessary files for performing alchemical MD simulations with GROMACS.

**Figure 2:**
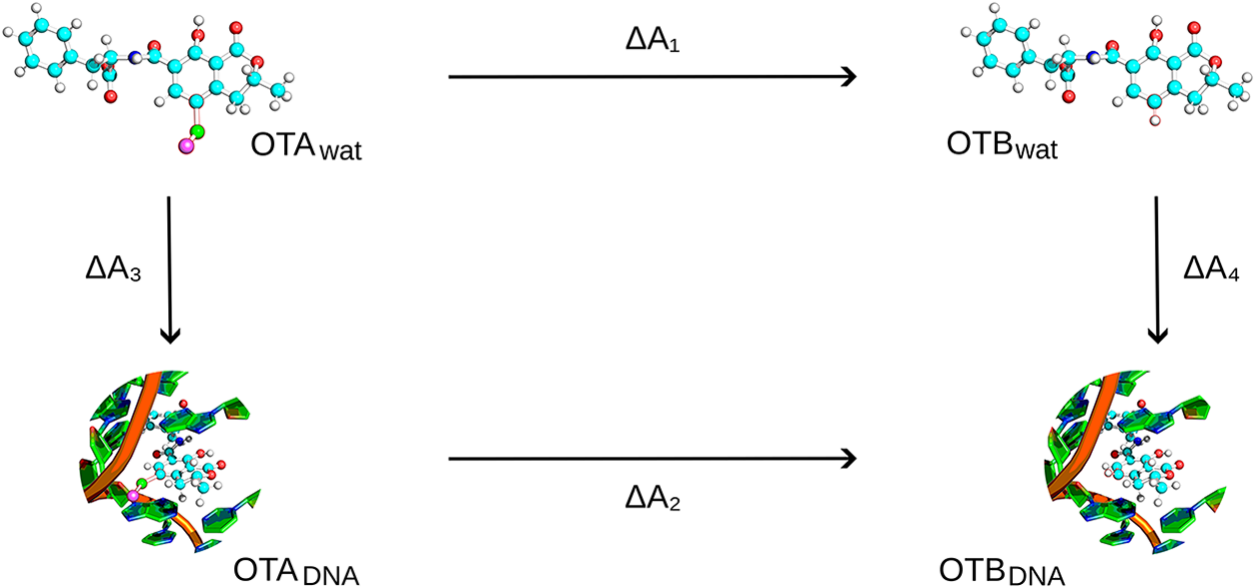
Representation of the thermodynamic cycle used to calculate relative binding free energies of the two ochratoxins (ΔΔ*A* = Δ*A*_4_ – Δ*A*_3_). Pmx-generated hybrid ligand structure for the OTA (λ = 0) to OTB (λ = 1) transitions are shown. Vertical arrows describe the binding process of the ligands to the aptamer. Horizontal arrows indicate the nonphysical pathways used in the current study, along which OTA and OTB were alchemically morphed.

### Alchemical Free Energy Calculations

The computational cost and complexity associated with determining the absolute binding free energies of OTA and OTB pose significant challenges. The relative binding Helmholtz free energy (ΔΔ*A*) between the two ligands bound to corresponding aptamer can be estimated using the thermodynamic cycle depicted in Figure 2. Subsequently, the relative binding free energy can be expressed in eq 1.

1 $$\Delta \Delta A=\Delta A_{4}-\Delta A_{3}=\Delta A_{2}-\Delta A_{1}$$

As shown in Figure 2, Δ*A*_1_ and Δ*A*_2_ describe the free energy change associated with the alchemical transformation of OTA into OTB in two different environments, namely, in water and in the aptamer binding pocket. Both values can be obtained through alchemical nonequilibrium free energy calculations using the fast growth method.^41,42,44,45^ Additional MD simulations were performed using the same set up described in the Molecular Dynamics Simulations section. The pmx-generated structure and topology for the hybrid ochratoxin were used as input files. For the two end states (λ = 0 and λ = 1) the system was prepared for forward and backward transition, respectively. After an initial energy minimization step, the systems were heated to 300 K for 1 ns. Position restraints were applied on the aptamer phosphorus atoms, with a force constant of 500 kJ mol^–1^ nm^–2^; this was followed by an unrestrained 5 ns equilibration. Accordingly, all subsequent simulations were performed without restraints. A production run of 20 ns was performed. Throughout this preparation phase, λ values were kept fixed (λ = 0 for OTA and λ = 1 for OTB). From each production trajectory, 100 snapshots were extracted (i.e., the systems were sampled every 200 ps); these served as starting points to perform nonequilibrium simulations. To ensure equilibrium of the initial velocities, a 20 ps run was performed for each structure at the corresponding fixed λ value. Finally, fast transitions, where λ was continuously changed from 0 to 1 (forward) or from 1 to 0 (backward), lasting 100 ps, were carried out. The soft-core potential was used for both Coulombic and van der Waals interactions, with values of α = 0.3 and σ = 0.25, and the soft-core power was equal to 1. For both forward and backward transitions, the λ parameter was adjusted at a constant rate of 2.0 × 10^–5^ per time step, ensuring completion of the alchemical transformation within the simulation time. The parameters were chosen according to those used by Gapsys and collaborators to perform fast transition dynamics.^42–44^ Simulations with two additional sets of parameters were performed to validate the obtained results (details in SI). The work performed on the system during these fast nonequilibrium transitions (W) can be interpreted as the energetic cost of changing the system’s Hamiltonian, *H*, and can therefore be evaluated by integrating the derivative of *H* with respect to the coupling parameter λ:

2 $$W=\int_{\lambda =0}^{\lambda =1}\frac{\delta H}{\delta \lambda }d\lambda$$

These derivatives were recorded at every step for each fast nonequilibrium simulation. The resulting work values from all of the transitions were then used to compute the forward and backward work distributions. The relative free energy difference, ΔΔ*A*, was determined using Crooks Gaussian Intersection (CGI), Bennett Acceptance Ratio (BAR) and Jarzynski (JE) estimators,^56–58^ based on the overlap of the calculated forward and backward work distributions. The whole procedure was replicated five times to estimate the mean value of ΔΔ*A* and its standard error. For all data analyses and ΔΔ*A* computations, the scripts from the pmx package were employed.^43^

## OBA3 Results

### Structure and Dynamics of OBA3

The tridimensional structure of the aptamer OBA3 in complex with OTA is characterized by a stable hairpin of the middle portion of the sequence (G5 to C11), which includes a GAA loop, and a triple helix consisting of both the terminal portions.^22^ The hairpin features two Watson–Crick GC base pairs (G5C11 and C6G10) and a noncanonical interaction between G7 and A9, two nucleobases of the loop. On the other hand, the triple helix is mainly stabilized by three GGC triplets (G14G2C18, G13G3C17 and G12G4C16) and a canonical GC interaction C1G19. Residues G4, G5, C11, G12, T15 and C16 constitute the binding pocket of OTA: further stabilization of the complex is due to hydrogen bonds with either amide proton or carbonyl oxygen of OTA, and stacking interactions with either isocoumarin or benzene ring of OTA. G5 is also believed to form halogen bonding with the chlorine atom of OTA. To check convergence of the MD simulations, the evolution of Root Mean Square Deviation (RMSD) of the phosphorus atoms of the aptamer with respect to the centroid of the NMR structure was evaluated, and reported in the SI (Figure S1). Additionally, the total number of hydrogen bonds was computed (Figure S2). The MD simulations of the ligand-free OBA3 aptamer revealed high conformational flexibility, consistent with experimental findings.^22^ Since the starting coordinates were based on a structure in which the aptamer engages in several interactions with OTA, a degree of mobility in the binding pocket was anticipated, particularly for the six nucleobases primarily involved in toxin recognition. Such a prediction was confirmed by RMSD of the phosphorus atoms of the aptamer (Figure S1). As demonstrated by the evaluation of the Root Mean Square Fluctuation (RMSF) for the entire aptamer with respect to the centroid of the NMR structures (Figure S3), the larger deviation of the aptamer structure for the ligand-free OBA3 compared to the OBA3 complexes is particularly evident for the G4, G5, C11 and T15 nucleobases, all involved in the toxin recognition. However, the flexibility of these residues often resulted in substantial reorganization of the overall DNA architecture, occasionally disrupting the characteristic triplets observed in the NMR models. An analysis of the hydrogen bonding network underlying the secondary structure of the aptamer throughout the simulations was performed. Selection of the interactions was based on the experimentally reported hydrogen bond network.^22^ The AT, AG, GC and GGC interactions were considered to be formed if at least 2, 2, 3, and 5 hydrogen bonds were identified, respectively; the criteria for assigning hydrogen bonding were a A–H distance below 3.5 Å and a A–H–D angle greater than 150°, where A is the acceptor and D the donor of the hydrogen bonding. The analysis revealed that while such nucleobase interactions remained largely conserved in both complexes, the ligand-free form rearrangements involved the loss of some of these contacts, as shown in Figure 3. Specifically, one of the five replicas of the ligand-free OBA3 underwent disruption of all three triplets (G14G2C18, G13G3C17 and G12G4C16). For all the other replicas, while such triplets were retained, the base pairings G5C11 and C6G10 were lost, and the G7A9 resulted generally less stable. To identify dominant motions, Principal Component Analysis (PCA) was performed on a combined trajectory including both the OTA-bound and ligand-free forms of the aptamer. To ensure statistical balance, the five simulations performed on free OBA3 were subsampled for the covariance analysis, therefore taking into account an equal number of conformations for the two aptamer forms. All simulations were then projected onto the first two principal components (PCs, which explain 54% of the total variance), as shown in Figure 4. Convergence of the simulations of OBA3-OTA and OBA3-OTB was further validated by the comparison of the projections of five consecutive blocks of the corresponding MD trajectories onto the 2D subspace described by the two first eigenvectors obtained by the PCA (Figure S4). The analysis highlights the enhanced flexibility of the studied sequence in the absence of the ochratoxin. Projections along the first two principal components show that, when in complex with either OTA or OTB, the aptamer explores a reduced conformational ensemble with respect to the ligand-free condition, where a wider region of the phase space is accessible and aptamer conformations can deviate substantially from the NMR structures resolved for the OBA3-OTA complex.

**Figure 3:**
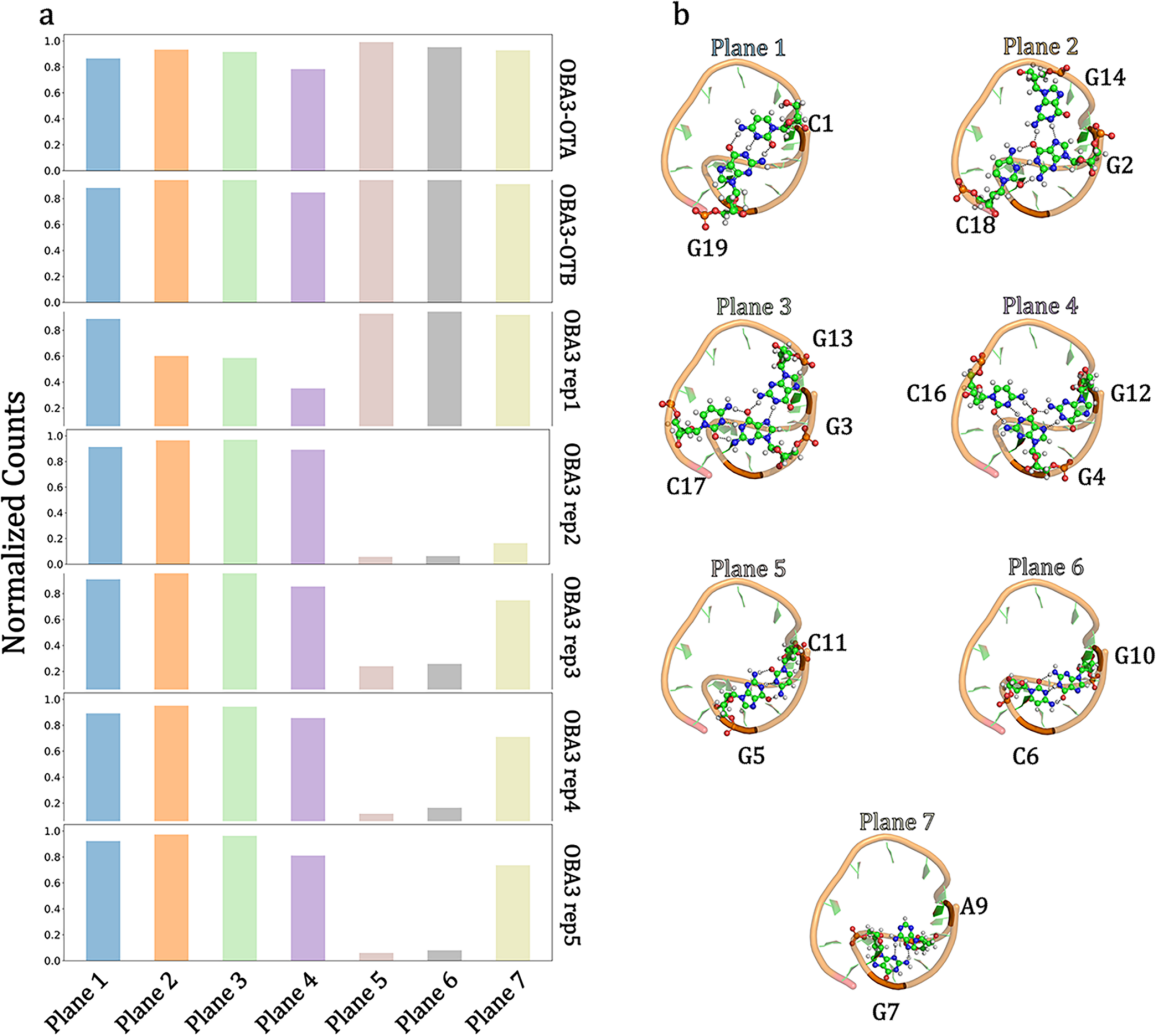
(a) Normalized counts of relevant hydrogen bonds among nucleobases, selected based on the NMR structure (PDB entry 6J2W) and monitored during the MD simulations, are reported for the ligand-free OBA3 system, the OBA3-OTA complex, and the OBA3-OTB complex. Each row represents a different system and each column a different aptamer plane. (b) Top views of the OBA3 aptamer highlight planes considered for the analysis. Details concerning the hydrogen donors/acceptors pairs are reported in Table S3.

**Figure 4:**
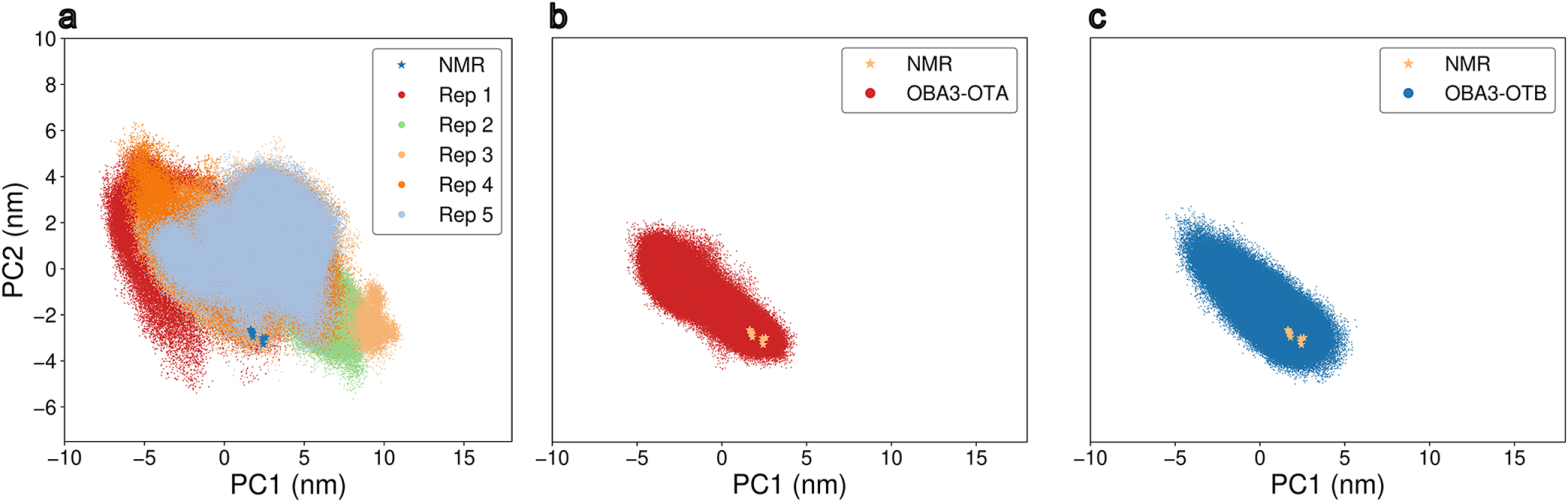
Projections of the MD trajectories on the 2D subspace described by the two first eigenvectors obtained by the PCA. Points represent different frames from the corresponding simulation. (a) ligand-free OBA3 across five MD simulations, colored by replica. Representative structures from each replica of the unbound system are shown. (b) OBA3-OTA. (c) OBA3-OTB.

These findings corroborate the experimental data, showing that the OBA3 system in the unbound form explores a large pool of different conformations along the molecular dynamics trajectory. Such observations support the hypothesis that ligand binding promotes structural organization and constrains aptamer dynamics.^22^

### Binding Pocket Architecture

To investigate the structural features underlying the different recognitions of OTA and OTB by the OBA3 aptamer, MD trajectories of both complexes were analyzed, focusing on hydrogen bonding and stacking interactions within the binding pocket, as shown in Figure 5. During the simulations, OTA predominantly formed a hydrogen bond network that closely resembles the E-model configuration proposed by Xu and collaborators,^22^ characterized by interactions between the carbonyl oxygen of OTA and the exocyclic amino hydrogens of guanine residue G12 and between the amide proton of OTA and the N3 atom of guanine residue G5. In contrast, the OTB-bound aptamer more frequently engaged in a network consistent with the D-model, showing a broader distribution of hydrogen bonding contacts, including those with exocyclic amino hydrogens of G5 and with the carbonyl oxygen of guanine G4. In terms of stacking interactions, OTA exhibited enhanced stacking with cytosine C11 and weaker stacking with guanine G5 and C16, whereas the opposite trend was observed for OTB. This pattern reflects a deeper placement of OTA within the binding pocket, supporting the idea of a higher affinity of the aptamer for this ligand. The stacking with guanine G12 appears to be largely conserved between the two complexes, suggesting a robust interaction that is not strongly influenced by variations in ligand positioning. Thymine T15 is also engaged in stacking interactions with both OTA and OTB, despite showing a higher distance for this contact than for the more planar π–π stacking interactions. This is consistent with a T-shaped stacking arrangement, which involves an edge-to-face geometry, typically weaker than parallel stacking.

**Figure 5:**
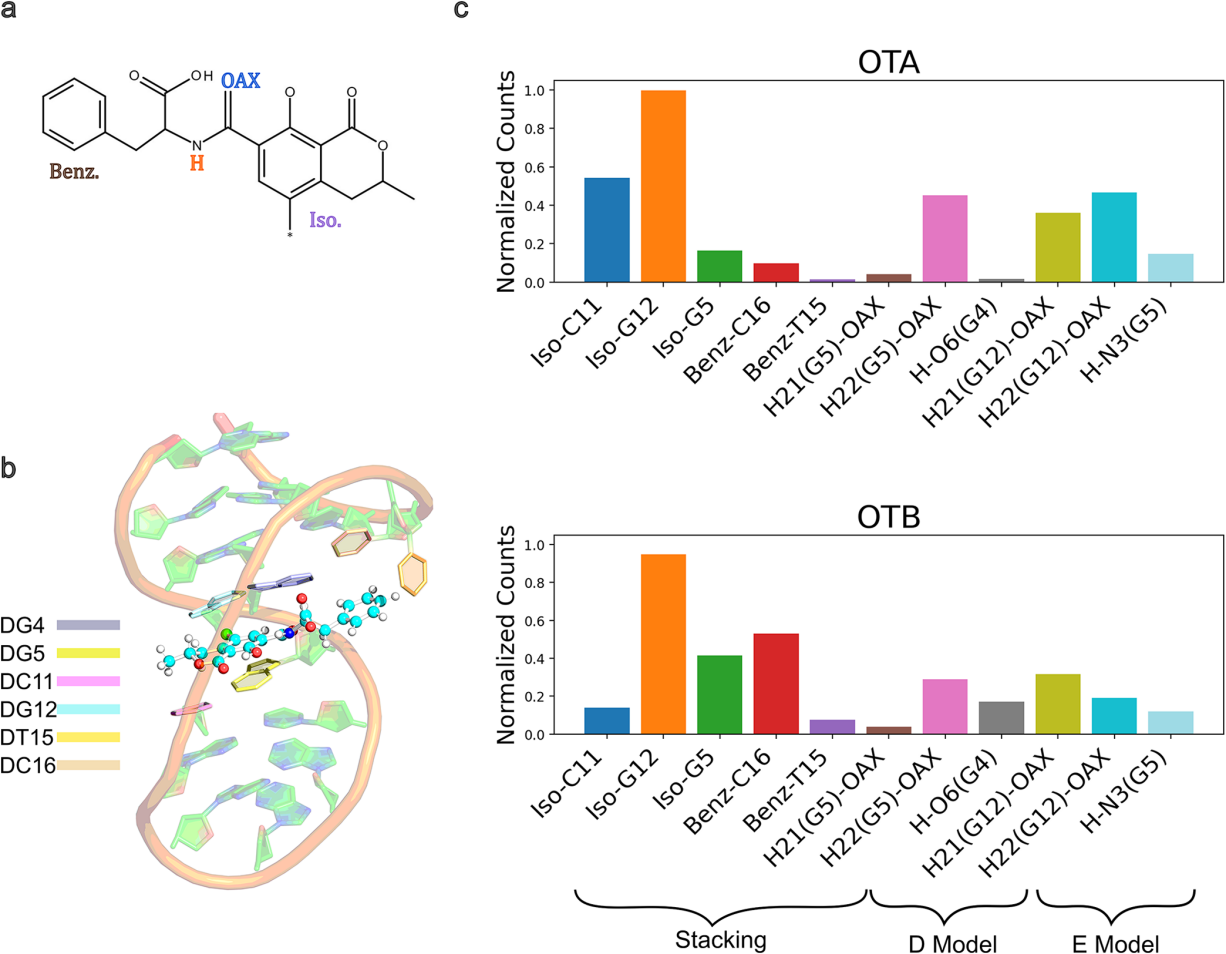
(a) Structure of ochratoxin (OTA or OTB); atoms and moieties involved in binding pocket interactions are highlighted. (b) Representative snapshot of the MD simulation of the OBA3-OTA complex. The binding pocket nucleobases are highlighted. (c) Comparison of relevant stacking interactions of isocoumarin ring (Iso.), benzene ring (Benz.) and hydrogen bonds of amide proton (H), amide carbonyl oxygen (OAX) of the ligands with the binding pocket nucleobases observed during the OBA3-OTA (top) and OBA3-OTB (bottom) MD simulations.

### Halogen Bond Analysis

Among the noncovalent interactions observed within the binding pocket, halogen bonding emerges as a unique feature of the OTA–aptamer complex, as it cannot occur in the OTB counterpart. While hydrogen bonding and stacking interactions are present in both systems, albeit with different patterns, the ability of OTA to engage in halogen bonding may represent a critical factor in selective recognition. To assess the relevance of this interaction, a dedicated analysis of the MD simulation was carried out, taking into account not only interatomic distances but also the orientation required for halogen bonds. Based on the structural insights provided by Xu and collaborators,^22^ the chlorine atom of OTA was hypothesized to form halogen bonds with electron-rich atoms on the guanine base at position 5, namely the atoms N7, N9, and O6. This hypothesis, based on NMR structural data, is founded on specific geometric conditions: a Cl-D distance below 4.2 Å and a C–Cl–D angle greater than 110°, where D denotes the electron donor atom. The choice for these thresholds is consistent with previous structural studies on the OTA–aptamer system.^22^ While more stringent criteria are sometimes employed in crystallographic or theoretical analyses, the values adopted here are appropriate for MD simulations, where structural fluctuations and conformational flexibility tend to broaden the distribution of distances and angles of halogen bond geometries.^59,60^ To assess the frequency and stability of these contacts throughout the simulation, combined distribution functions (CDFs) of distance and angle were computed for each of the three candidate donor atoms. The simulation model incorporated a positively charged dummy atom to emulate the σ-hole on chlorine, providing a more realistic representation of the anisotropic electrostatic potential responsible for halogen bonding. As shown in Figure 6, only the Cl–N7 interaction of guanine G5 consistently meets the geometric criteria over a significant portion of the simulation time (34.2% of the trajectory, with mean distance 0.36 nm and mean angle 107°), whereas contacts with N9 and O6 were rarely observed (0.8% and 0.3%, with mean distances 0.45 and 0.38 nm and mean angles 88° and 83°, respectively).

**Figure 6:**
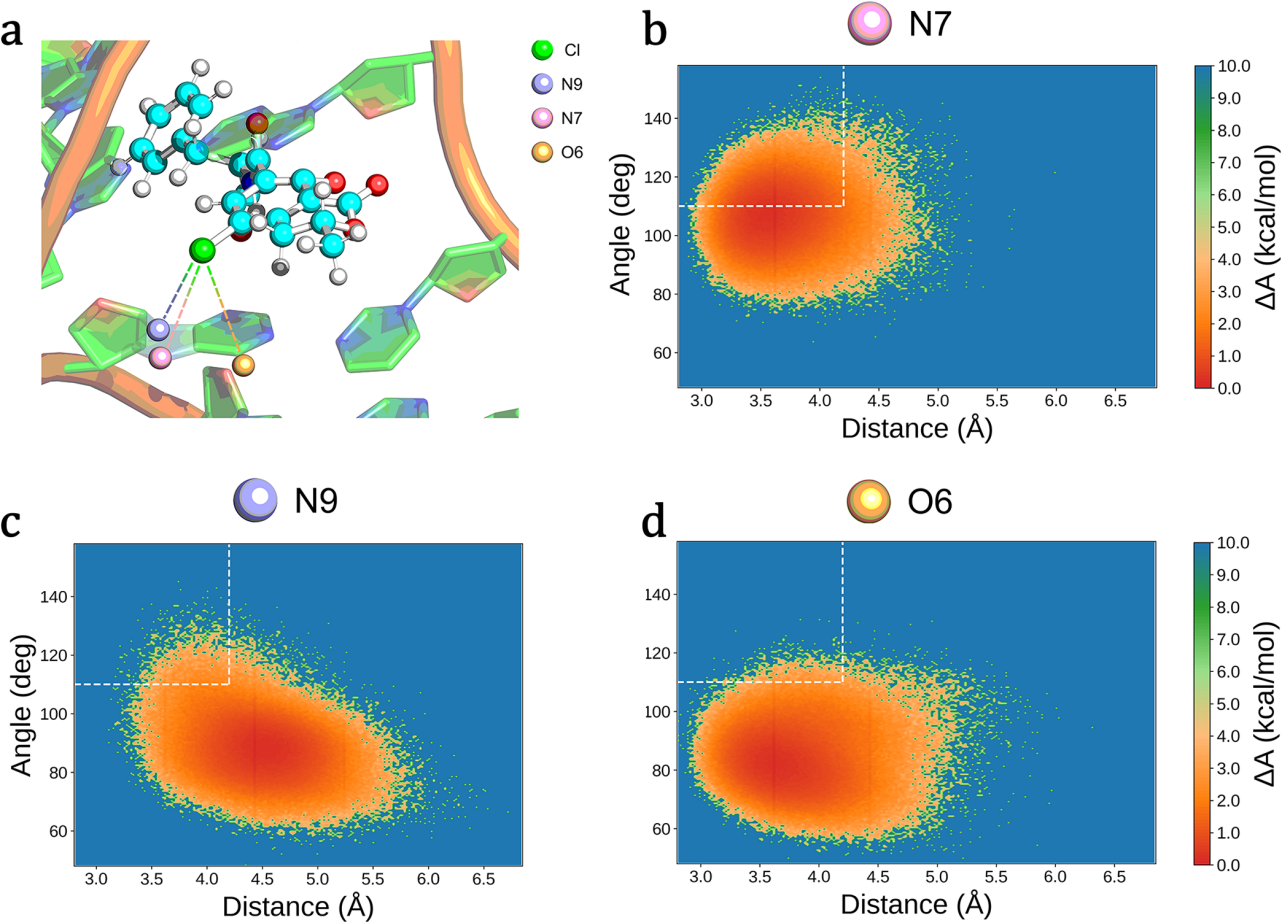
(a) Expanded view of OTA in the binding pocket of OBA3; the potential halogen bonds are highlighted (colored dashed lines). Combined distribution functions for Cl-D distance and C–Cl–D angle (where D is the donor of electron density) are shown for N7 (b), N9 (c), and O6 (d) atoms of G5 to evaluate halogen interactions. Dotted lines show the threshold values for distances and angles.

This suggests that the Cl–N7 interaction plays a dominant role in stabilizing the OTA–aptamer complex. These results align well with the NMR-derived hypothesis concerning halogen bonds and further reinforce the importance of such interaction as a key determinant in the selective recognition of OTA. The presence of halogen bonding in the OBA3–OTA complex also provides an explanation for the distinct stacking patterns relative to the OBA3–OTB complex (Figure 5). In the OTB complex, the isocoumarin ring engages in stable π–π stacking with guanine G5; on the other hand, OTA adopts a binding geometry which better satisfies the constraints required for halogen bonding, particularly the large C–Cl–N7 angle. This orientation promotes halogen bond formation but comes at the expense of optimal π–π stacking with guanine G5. As a result, OTA occupies a deeper position within the binding pocket than OTB, and forms stronger stacking interactions with the C11 base, which better accommodates the halogen-bond compatible geometry. In contrast, OTB, which lacks the chlorine substituent and the consequent directional constraints, remains closer to the peripheral region of the pocket and preferentially maximizes π–π stacking with both G5 and C16. Interestingly, during the simulation, the possibility of an additional halogen bond, between the chlorine atom and the O2P oxygen atom of the phosphodiester bridge between residues G3 and G4, emerged. This Cl–O2P contact met the geometric criteria for halogen bonding assignment during a substantial portion of the trajectory (51.2%), with a mean distance of 0.43 nm and a mean angle of 153° (Figure S5). The interaction occurred primarily following a slight displacement of OTA within the binding pocket, approximately parallel to the base plane. Although this contact was not observed in the reported NMR-based structures, its presence in the simulation raises the possibility that it could also be involved in further stabilizing the OTA–aptamer complex.

### Free Energy Difference Between OBA3-OTA and OBA3-OTB

Despite extensive sampling through classical MD simulations, it was not possible to detect the detachment process for either of the two ochratoxins. This outcome was anticipated for OTA; however, experimental estimates indicate a weak binding affinity for OTB.^22^ Despite only weakly binding to the aptamer, unbinding events of OTB may still occur on time scales beyond those accessible to unbiased MD within practical computational times. Therefore, the determination of the affinity of OTA and OTB toward OBA3 was achieved using alchemical methods for the calculation of the free energy difference (eqs 1 and 2). This choice was motivated by previous attempts using SMD and US that were unable to yield reliable estimates (data not shown). As detailed in the Alchemical Free Energy Calculations section, five independent hybrid systems were built. Using these inputs, five independent calculations were performed to estimate the free energy difference in water (Δ*A*_1_) and in OBA3 (Δ*A*_2_) that describe the alchemical transformation of OTA into OTB for each replica. Estimates were obtained by combining forward and backward work distributions with the BAR estimator, as shown in Figure 7. The resulting ΔΔ*A* values for each replica are reported in Table 1. Convergence was assessed by monitoring the mean relative binding free energy as the trajectory ensemble was progressively expanded, adding 100 trajectories at each step to the existing data set (data not shown). Additional calculations were performed using the CGI and JE estimators (Table S4). These results align well with the experimental findings, confirming the higher affinity of OTA with respect to OTB toward OBA3 aptamer.^22^ Histograms of the work, reported in the SI (Figure S6) confirm satisfactory overlap for each replica. Additionally, the same calculations were performed for two more sets of switching parameters. The results (Tables S5 and S6) and work histograms (Figures S7 and S8) validated the estimated free energy difference between the two ochratoxin-aptamer complexes.

**Figure 7:**
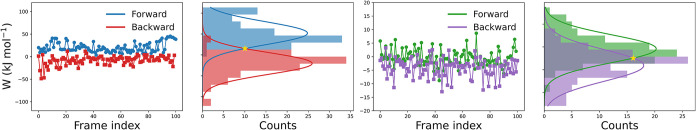
Calculated work values (W) for each extracted frame in the alchemical free energy calculations of one replica, shown in OBA3 (left) and in water (right) systems. Forward (blue/green circles) and backward (red/purple squares) work distributions are plotted, along with their respective histograms. The BAR integrator was used to estimate the free energy differences indicated by the asterisk on the histogram overlap.

**Table 1:** Free Energy (kcal mol^–1^) Difference Obtained for Each Replica^a^

rep.	ΔΔ*A*
I	2.0
II	2.9
III	2.7
IV	3.5
V	3.7
mean	2.9 ± 0.5

a ΔΔ*A*: relative free energy of binding for OBA3 using the BAR integrator.

## OBA33 Results

### Structure and dynamics of OBA33

The OBA33 aptamer in complex with OTA adopts a duplex-quadruplex structure. The duplex portion is stabilized by four Watson–Crick interactions, namely, C1G33, G2C32, A3T31 and T4A30. Two guanine tetrads (G7G26G16G13 and G8G12G17G25) are involved in G-quadruplex interactions. The structure is also characterized by three loops, namely T9 to T11, G14T15 and C18 to G24; hydrogen bond interactions are observed among the nucleobases of these loops, including a G10G24C18 triplet.^23^ The binding pocket of OTA is located in the junction between the duplex and quadruplex regions; the toxin forms stacking interactions with T4, A30 and G28 via its isocoumarin ring and with G28 and A27 via its benzene ring, as well as a hydrophobic interaction between the methyl group and the C29 base. Finally, OBA33 can form a halogen bond with the chlorine atom of OTA, similarly to OBA3. However, in this case, the chlorine interacts with the pyrimidine ring of the C5 nucleobase. Time evolution of RMSD of phosphorus atoms of the aptamer with respect to the centroid of NMR structures (PDB: 7W9N) and total number of hydrogen bonds within the aptamer were inspected to verify convergence of the simulations, and are reported in the SI (Figures S9 and S10). These analyses suggest that for both complexes the aptamer structure and interactions were mostly conserved, as the RMSD values and number of hydrogen bonds are reasonably stable. On the other hand, the five ligand-free simulations showed an increased flexibility: for three of the replicas, both RMSD and number of hydrogen bonds exhibited significantly reduced stability. However, the other two replicas proved to be more stable. These results suggest that OBA33 is characterized by an enhanced stability compared to that of OBA3. An analysis of the hydrogen bonds among selected nucleobases was performed in order to identify whether such interactions were conserved throughout the MD simulations, as shown in Figure 8. Selection of the interactions was based on the experimentally reported hydrogen bonding network.^23^ The criteria for assigning hydrogen bonds were the same as those described for the analysis in OBA3 (A–H distance, A–H–D angle, and formation of GC, AT, AG and GGC interactions). Additionally, the formation of G-quadruplex structures and GT pairing were considered when at least 7 and 2 hydrogen bonds were observed, respectively.

**Figure 8:**
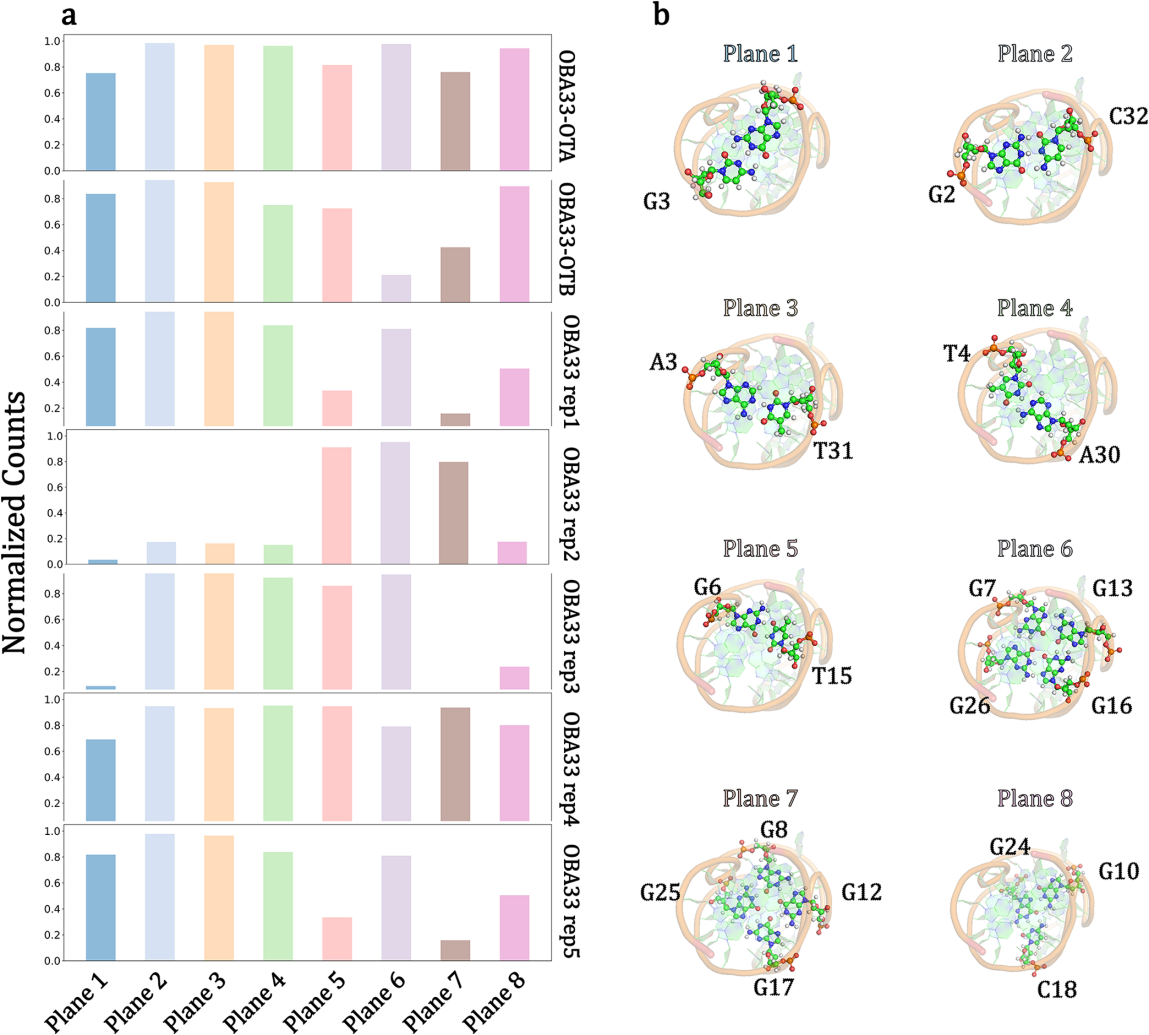
(a) Normalized counts of relevant hydrogen bonds among nucleobases, selected based on the NMR structure (PDB entry 7W9N) and monitored during the MD simulations, are reported for the ligand-free OBA33 system, the OBA33-OTA complex, and the OBA33-OTB complex. Values are divided by aptamer plane. (b) Top views of the OBA33 aptamer highlight planes and hydrogen bonds considered for the analysis. Details concerning the hydrogen donors/acceptors pairs are reported in Table S7.

The four Watson–Crick base pairings of the duplex region, as well as the noncanonical G6T15 interaction, the two guanine tetrads involved in G-quadruplex interactions, and the GGC triplet were analyzed. Results showed that while such interactions were largely retained for the OBA33-OTA complex, they were variably disrupted across replicas in the ligand-free simulations. Specifically, the duplex region base pairings were lost in one of the simulations, the GGC triplet showed a general decrease in stability, and one of the G-quadruplex structures was totally disrupted in two replicas. Finally, the OBA33-OTB complex retained both its duplex structure and the GGC triplet, while showing weaker G-quadruplex interactions relative to those of OBA33-OTA, indicating a reduced stabilizing effect of OTB compared to that of OTA. To further verify the increased flexibility of the ligand-free system, PCA was performed on the atoms of the concatenated trajectory of the OBA33-OTA complex and the five ligand-free simulations. Each of the 5 replicas was subsampled to ensure statistical balance, adopting the same procedure described for the OBA3 aptamer PCA. Projections of all trajectories along the first two principal components, constituting 48% of the total variance, are shown in Figure 9. Similarly to what was observed for the OBA3 system, the ligand-free OBA33 aptamer explored a significantly wider region of the reduced phase space if compared with the aptamer bound to each of the toxins. Additionally, convergence of the simulations of OBA33-OTA and OBA33-OTB was further validated by comparing the projections of five consecutive blocks of the corresponding MD trajectories on the 2D subspace described by the first two eigenvectors obtained by the PCA (Figure S11).

**Figure 9:**
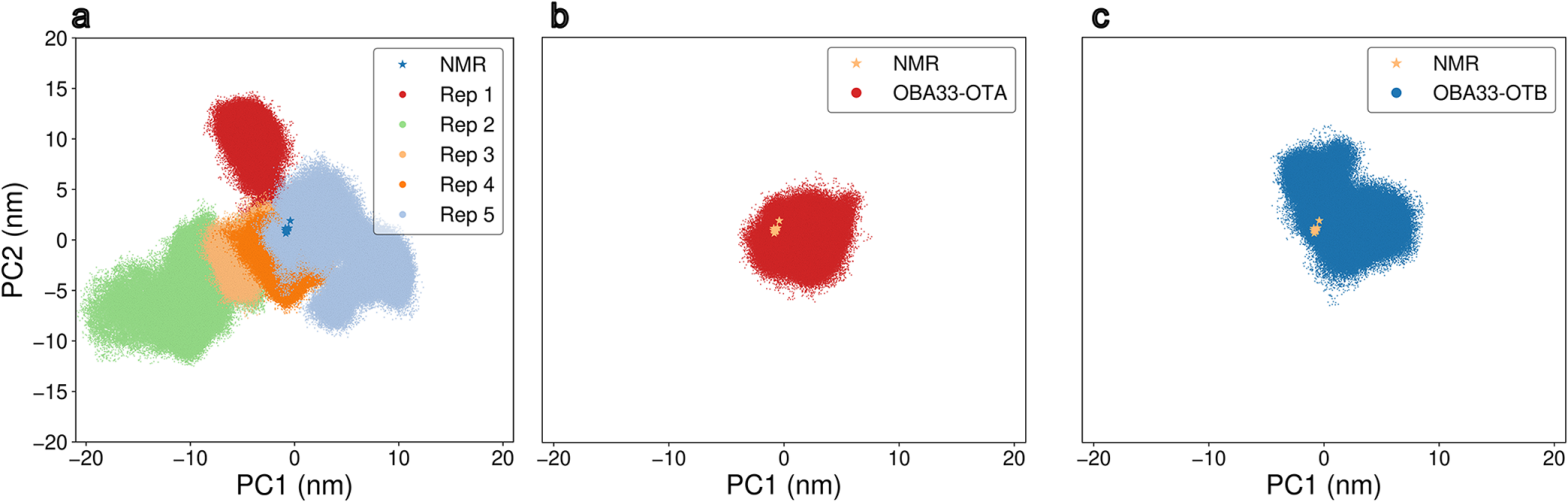
Projections of the MD trajectories on the 2D subspace described by the two first eigenvectors obtained by the PCA. Points represent different frames from the corresponding simulation. (a) ligand-free OBA33 across five MD simulations, colored by replica. (b) OBA33-OTA. (c) OBA33-OTB.

### Binding Pocket Architecture

The binding pocket of the OBA33 aptamer comprises the nucleobases C5, T4, A27, G28, C29 and A30. While OTA is mainly anchored to the aptamer by the halogen bonding between its chlorine atom and the aromatic ring of C5, the other nucleobases contribute to the stabilization of the complex via stacking and hydrophobic interactions with OTA.^23^ To investigate the differences in such binding patterns between OTA and OTB, these interactions were analyzed in the MD simulations of the OBA33-OTA complex and the OBA33-OTB complex (Figure 10) and compared with the reported experimental results. Even though NMR structures hint to hydrophobic contacts between the benzene ring of the toxins and the sugar moieties of A27 and G28, MD simulations showed no relevant sampling of conformations compatible with these interactions. Conversely, the analysis confirmed a pronounced stacking interaction between the isocoumarin ring of both OTA and OTB and the nucleobase G28, together with a certain degree of stacking interaction with the T4A30 base pair and a hydrophobic interaction between the toxins’ methyl group and the C29 nucleobase, as reported experimentally. Additionally, two hydrogen bonds, not reported in the experimental structures, were observed. Overall, these interactions appeared more persistent in OBA33-OTA compared to OBA33-OTB, substantiating the selectivity of the proposed sequence among the two analogues.

**Figure 10:**
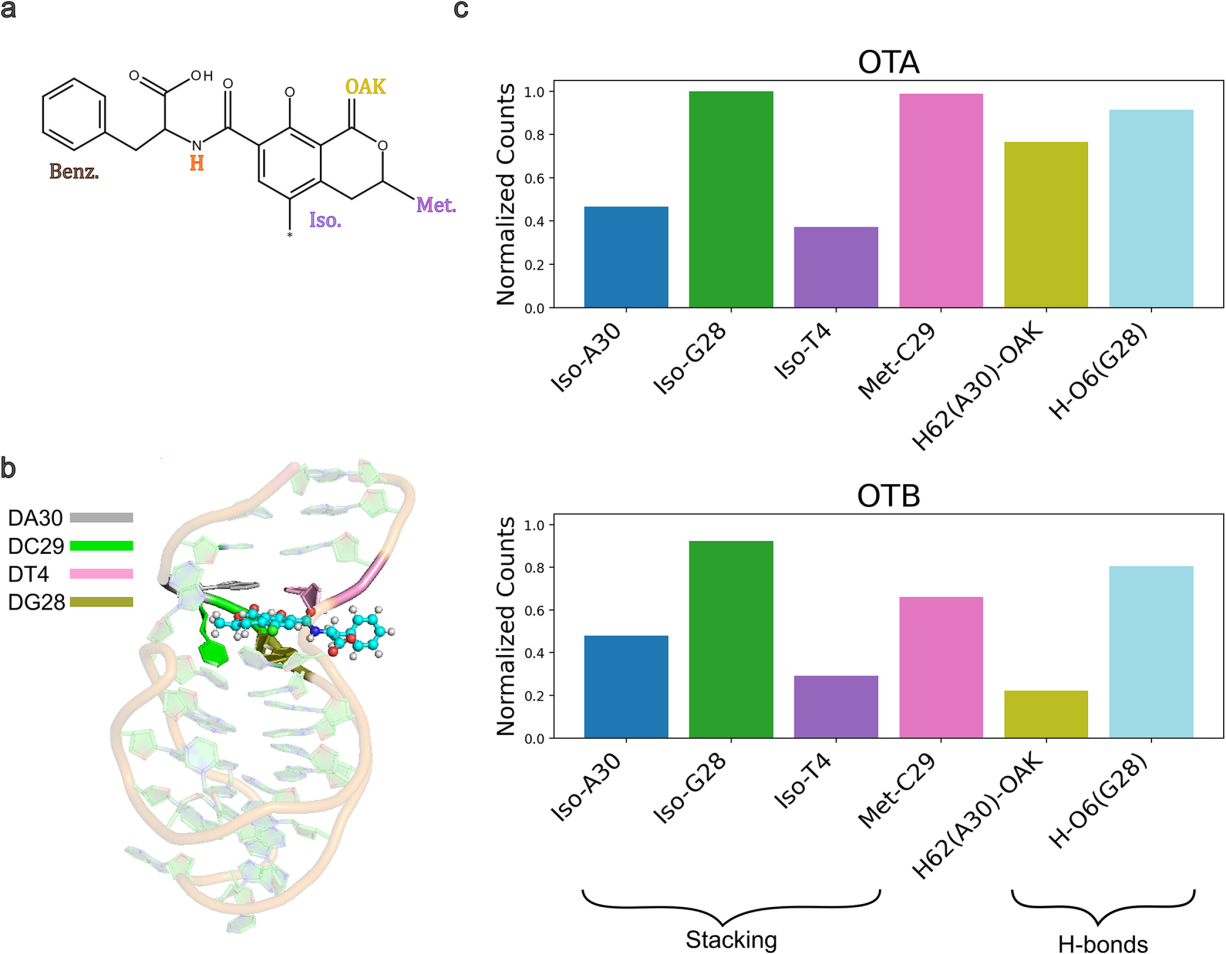
(a) Structure of ochratoxin (OTA or OTB); atoms and moieties involved in binding pocket interactions are highlighted. (b) Representative snapshot of the MD simulation of the OBA33-OTA complex. The binding pocket nucleobases are highlighted. (c) Comparison of relevant stacking interactions of isocoumarin ring (Iso.), hydrophobic interaction of the methyl group (Met.) and hydrogen bonds of amide proton (H), lactone carbonyl oxygen (OAK) of the ligands with the binding pocket nucleobases observed during the OBA33-OTA (top) and OBA33-OTB (bottom) MD simulations.

### Halogen Bond Analysis

A more detailed analysis was performed to evaluate the persistence of the halogen bond between the chlorine atom of OTA and the pyrimidine ring of the C5 of the OBA33 aptamer.^23^ The formation of this bond was evaluated according to the geometric criteria previously adopted for the OBA3 sequence (Cl-D distance and C–Cl–D angle, where D is the center of mass of the heavy atoms of the pyrimidine ring of the C5 base). The obtained results showed a remarkable stability of the halogen bond, sampled over more than 88% of the simulation time, with an average distance of 3.6 Å and average angle of 128°. The combined distribution function for the Cl–C5 distance and C–Cl–C5 angle is shown in Figure 11.

**Figure 11:**
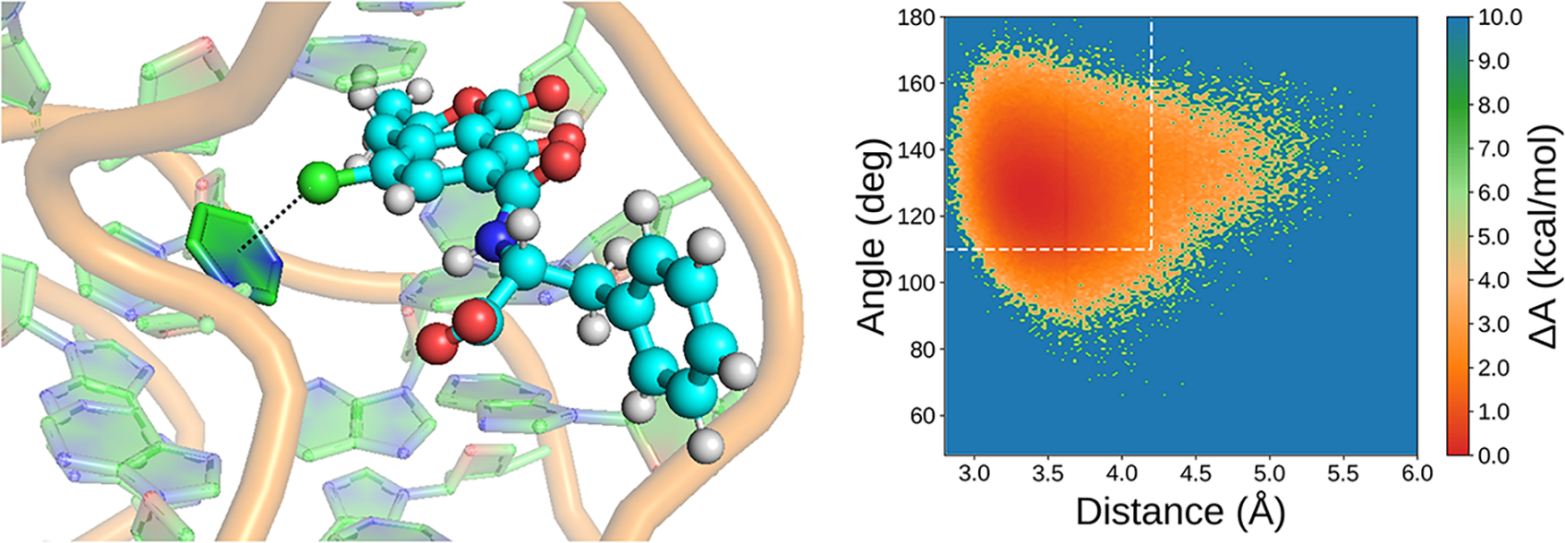
On the left, expanded view of OTA in the binding pocket of OBA33. The potential halogen bond is highlighted (dashed line). On the right, combined distribution function for Cl–C5 distance and C–Cl–C5 angle to evaluate halogen interaction. Dotted lines show the threshold values for distances and angles.

### Free Energy Difference Between OBA33-OTA and OBA33-OTB

As previously reported for OBA3, it was not possible to detect the detachment process by means of classical MD simulations for either of the two ochratoxins. The modeling of the selectivity of OBA33 toward OTA over OTB was achieved using alchemical methods for the calculation of free energy difference (eqs 1 and 2) . As detailed in the Alchemical Free Energy Calculations section, five independent hybrid systems were built. Using these inputs, five independent calculations were performed to estimate the free energy difference in water (Δ*A*_1_) and in OBA33 (Δ*A*_2_) that describe the alchemical transformation of OTA into OTB for each replica. Estimates were obtained by combining forward and backward work distributions with the BAR estimator, as shown in Figure 12. The corresponding values for each estimator are listed in Table 2. Satisfactory overlap is shown by histograms of the work reported in the SI (Figure S12) for each replica. Additional calculations were performed using the CGI and JE estimators (Table S8). For the OBA33 system, binding free energies were successfully estimated experimentally and reported for both ochratoxin-aptamer complexes. The difference between these two values is approximately 2.7 kcal mol^–1^, in rather good agreement with the results obtained in this study.^23^

**Figure 12:**
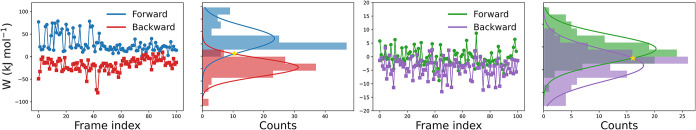
Calculated work values (W) for each extracted frame in the alchemical free energy calculations of one replica, shown in the OBA33 (left) and water (right) systems. Forward (blue/green circles) and backward (red/purple squares) work distributions are plotted along with their respective histograms. The BAR integrator was used to estimate the free energy differences, indicated by the asterisk on the histogram overlap.

**Table 2:** Free Energy (kcal mol^–1^) Difference Obtained for Each Replica^a^

rep.	ΔΔ*A*
I	2.2
II	1.9
III	2.2
IV	2.6
V	1.9
mean	2.2 ± 0.3

a ΔΔ*A*: relative free energy of binding for OBA33 using the BAR integrator.

## Conclusion

In light of the growing interest in aptamers as versatile agents for molecular recognition, there is an increasing need to establish standardized procedures for determining the aptamer-target binding affinity and specificity. To date, several factors have been reported to affect the reproducibility of such estimates, and MD simulations clearly offer a valuable complementary strategy. Building on this idea, our goal was to assess the potential of MD simulations in aptamer research using the OTA binding aptamers as a case study. We first performed classical MD simulations on the unbound aptamers and on their complexes with OTA and OTB. The resulting conformational ensembles enabled a detailed structural characterization of these systems, providing insights into the dynamics of the examined sequences in the absence and presence of the ligand as well as a comprehensive analysis of the key interactions within the binding pocket that stabilize the complexes at the atomic level. Notably, we introduced a dummy atom that models the chlorine anisotropic charge distribution in OTA to explicitly include the key halogen interactions. MD trajectory analyses supported the formation of such interactions. Our comparison of interaction patterns highlighted subtle differences between OTA and OTB binding, which offered valuable insights into the molecular basis of selective recognition. We then aimed to provide thermodynamic characterization of the complexes. To this end, we employed fast nonequilibrium transitions to estimate the relative affinities of OBA3 and OBA33 for OTA and OTB. The halogen bonds were modeled in the free energy calculations using the same approach as in the classical MD simulations. The method correctly predicted the specificity of both aptamers toward OTA, their primary target, and provided an estimate of the relative free energy difference between the two corresponding complexes. To the best of our knowledge, this is the first study applying such an approach to model aptamer binding. Importantly, the method yielded reproducible results across multiple replicas, in close agreement with the experimental estimates. In summary, these computational methods show promise for binding affinity estimation when a starting experimentally determined structure for the analyzed system is available. Such results can represent complementary tools to support the improvement of standardized procedures for the validation of newly developed aptamer-based systems.

## Supplementary Material



## Data Availability

All the files required to reproduce the results of this work are openly available at the following Zenodo repository https://zenodo.org/records/18199595.

## Notes

Comparison between ACPYPE partial charges and ESP charges for OTA at the HF/6–31G­(d) level of theory (with and without dummy atom). Halogen bond binding energies for the OTA-pyrimidine complex evaluated by both QM calculations and MM force field, and comparison with literature data. RMSD time evolution and distribution, total number of hydrogen bonds and RMSF profiles for OBA3 in the ligand-free state and in complex with the two ochratoxins. RMSD time evolution and distribution, total number of hydrogen bonds for OBA33 in the ligand-free state and in complex with the two ochratoxins. List of the donors/hydrogens/acceptors evaluated for selected interbase hydrogen bonds analyses of OBA3 and OBA33. Projections of the trajectories of the OBA3 and OBA33 complexes along the first two eigenvectors of the PCA analyses, divided into blocks of the simulation. CDF of distance and angle for the Cl–O2P halogen bond contact. Alchemical free energy diagnostics for the OBA3 and OBA33 systems. (PDF)
