# Supplementary material for: Assessing Molecular Dynamics in Predicting Aptamer–Ligand Binding Thermodynamics: Insights from the OTA Binding Aptamers
Source: J Chem Inf Model. 2026 Apr 3;66(8):4744–57. doi: 10.1021/acs.jcim.5c02643 (PMC13126630; doi:10.1021/acs.jcim.5c02643)
Supplement: Supplementary file 1 [file ci5c02643_si_001.pdf]

Supporting Information: Assessing Molecular Dynamics in Predicting Aptamer–Ligand Binding Thermodynamics: Insights from the OTA Binding Aptamers

Alessio Olivieri,[†,‡] Federica Borzelli,[†,‡] Mauro Giustini,[†] and Marco D'Abramo[*,†]

†*Department of Chemistry, Sapienza University of Rome, 00185 Rome, Italy*

‡*These authors contributed equally to this work*

E-mail: marco.dabramo@uniroma1.it

Comparison of the Calculated Charges for OTA

Table S1: Comparison between the ESP charges (e) at the HF/6-31G(d) level of theory and dummy atom corrected ACPYPE charges.

Atom	HF/6-31G(d)	HF/6-31G(d) with dummy	ACPYPE with dummy
C	-0.259241	-0.253137	-0.127500
C	-0.110958	-0.099425	-0.144500
C	-0.202457	-0.190976	-0.152000
C	-0.121018	-0.135234	-0.144500
C	-0.248487	-0.212865	-0.127500
C	0.199784	0.182110	-0.058300
C	-0.287657	-0.258413	-0.022100
C	0.283153	0.470557	-0.069300
C	0.757616	0.707295	0.902603
O	-0.780157	-0.768254	-0.834801
O	-0.764073	-0.761809	-0.834801
N	-0.743214	-0.793879	-0.483900
C	0.937279	0.899882	0.667701
O	-0.658616	-0.668530	-0.581101
C	-0.518657	-0.450078	-0.188600
C	0.212628	0.127354	-0.023000
C	-0.301247	-0.233527	-0.039600
Cl	-0.095606	-0.138687	-0.128600
C	0.445341	0.465672	-0.001300
C	-0.361202	-0.411370	-0.079100

Continued on next page

Table continued from previous page

Atom	HF/6-31G(d)	HF/6-31G(d) with dummy	ACPYPE with dummy
C	0.696379	0.666622	0.149100
C	-0.483649	-0.510475	-0.096100
O	-0.601164	-0.593209	-0.414900
C	1.016708	1.041759	0.752201
O	-0.661157	-0.660506	-0.625501
C	-0.758540	-0.794097	-0.286600
C	0.703374	0.683322	0.253100
O	-0.646610	-0.625605	-0.471100
H	0.193162	0.184351	0.159000
H	0.114882	0.107540	0.114500
H	0.122802	0.114448	0.109000
H	0.112493	0.111389	0.114500
H	0.169797	0.141908	0.159000
H	0.083349	0.055097	0.051200
H	0.093914	0.074756	0.051200
H	0.015299	-0.045836	0.082700
H	0.349899	0.356407	0.286500
H	0.109152	0.138828	0.237000
H	0.114908	0.130466	0.076200
H	0.080448	0.098573	0.076200
H	-0.063569	-0.044266	0.056700
H	0.126500	0.139022	0.048033
H	0.130243	0.115880	0.048033

Continued on next page

Table continued from previous page

Atom	HF/6-31G(d)	HF/6-31G(d) with dummy	ACPYPE with dummy
H	0.106054	0.138803	0.048033
H	0.492114	0.498133	0.454000
EP	–	0.038200	0.038200

Halogen Bond Binding Energy Validation

Calculated binding energy at the MM level of theory and at the MPWLYP/6-311+G(p,d).[1]

Table S2: Comparison of the MM and QM Halogen Bond Binding Energies (kcal mol^{-1}) for the OTA-pyridine (this work) and the chlorobenzene-pyridine complexes.[1]

	QM	MM
This Work	-0.95	-1.20
Ibrahim M.	-0.86	-0.95

OBA3

Structural Analysis

Table S3: Definition of the hydrogen bonds used for the OBA3 hydrogen bond analysis described in Figure 3.

Donor		Hydrogen		Acceptor	
Base	Atom	Base	Atom	Base	Atom
DC1	N4	DC1	H41	DG19	O6
DG2	N1	DG2	H1	DC18	N3
DG2	N2	DG2	H21	DC18	N3
DG2	N2	DG2	H21	DC18	O2
DG3	N1	DG3	H1	DC17	N3
DG3	N2	DG3	H21	DC17	N3
DG3	N2	DG3	H21	DC17	O2
DG4	N1	DG4	H1	DC16	N3
DG4	N2	DG4	H21	DC16	N3
DG4	N2	DG4	H21	DC16	O2
DG5	N1	DG5	H1	DC11	N3
DG5	N2	DG5	H21	DC11	O2
DC6	N4	DC6	H41	DG10	O6
DG7	N2	DG7	H22	DA9	N7
DA9	N6	DA9	H62	DG7	N3
DG10	N1	DG10	H1	DC6	N3
DG10	N2	DG10	H21	DC6	O2
DC11	N4	DC11	H41	DG5	O6
DG12	N1	DG12	H1	DG4	N7
DG12	N2	DG12	H21	DG4	O6
DG13	N1	DG13	H1	DG3	N7
DG13	N2	DG13	H21	DG3	O6
DG14	N1	DG14	H1	DG2	N7
DG14	N2	DG14	H21	DG2	O6
DC16	N4	DC16	H41	DG4	O6
DC17	N4	DC17	H41	DG3	O6
DC18	N4	DC18	H41	DG2	O6
DG19	N1	DG19	H1	DC1	N3
DG19	N2	DG19	H21	DC1	N3
DG19	N2	DG19	H21	DC1	O2

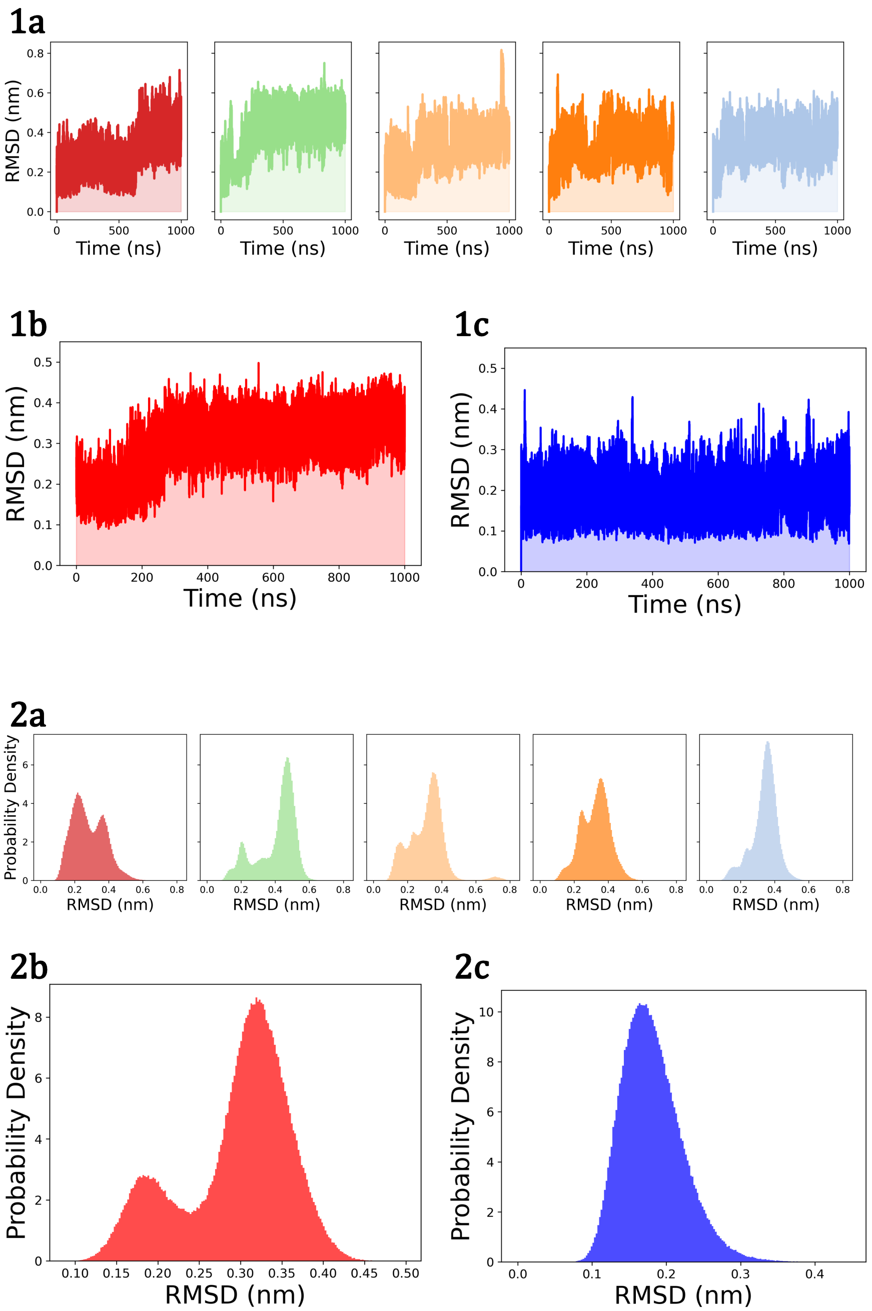

Figure S1: Root Mean Square Deviations computed on the phosphorous atoms of the OBA3 aptamer, with respect to the centroid of the models in PDB (6J2W). Both evolution in time and distributions ar shown. Panels: (1a) ligand-free OBA3 (5 replicas), 1b) OBA3-OTA complex, 1c) OBA3-OTB complex, 2a) distribution of ligand-free OBA3 RMSD (5 replicas), 2b) distribution of OBA3-OTA complex RMSD, 2c) distribution of OBA3-OTB complex RMSD.

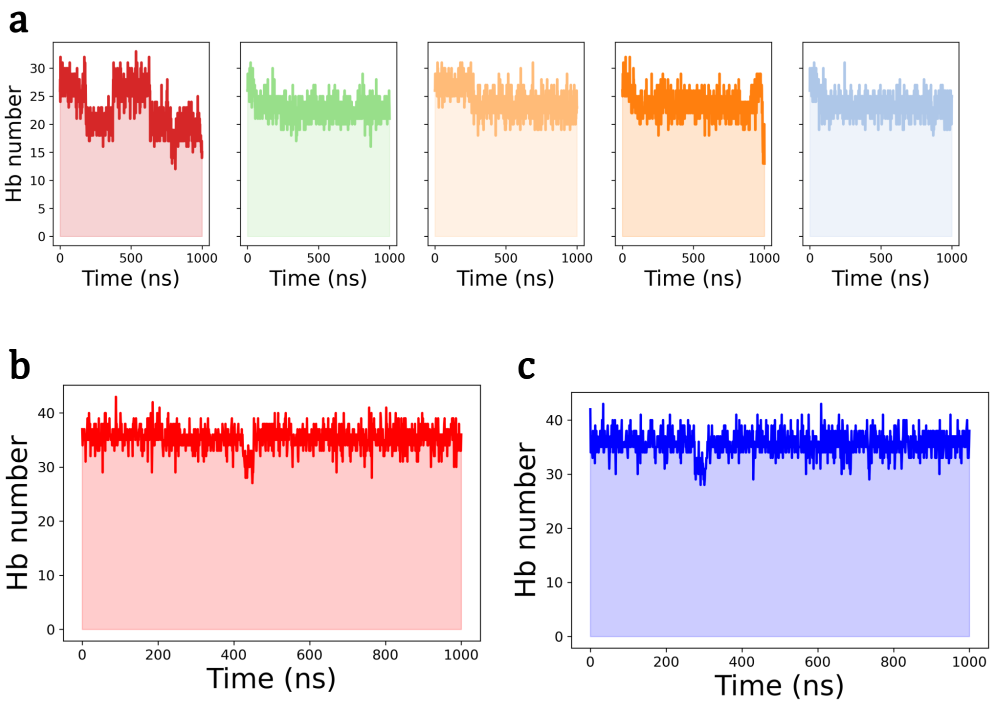

Figure S2: Hydrogen bonds sampled among nucleobases of the OBA3 aptamer throughout the simulation for (a) the 5 replicas of the ligand-free OBA3, (b) the OBA3-OTA complex, (c) the OBA3-OTB complex.

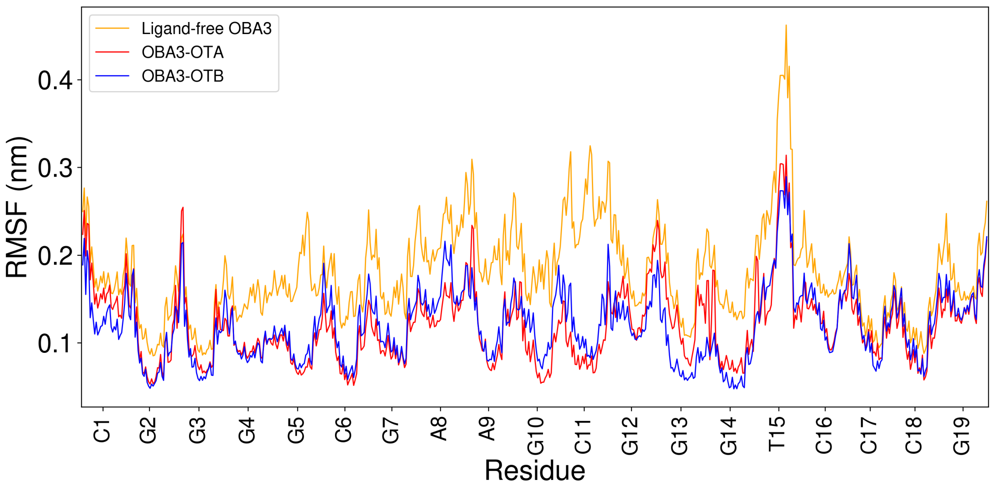

Figure S3: RMSF evaluated on all the atoms of the aptamer OBA3, using as reference structure the centroid of the models in PDB (6J2W), for the ligand-free OBA3 averaged over the 5 replicas (orange) the OBA3-OTA complex (red) and the OBA3-OTB complex (blue).

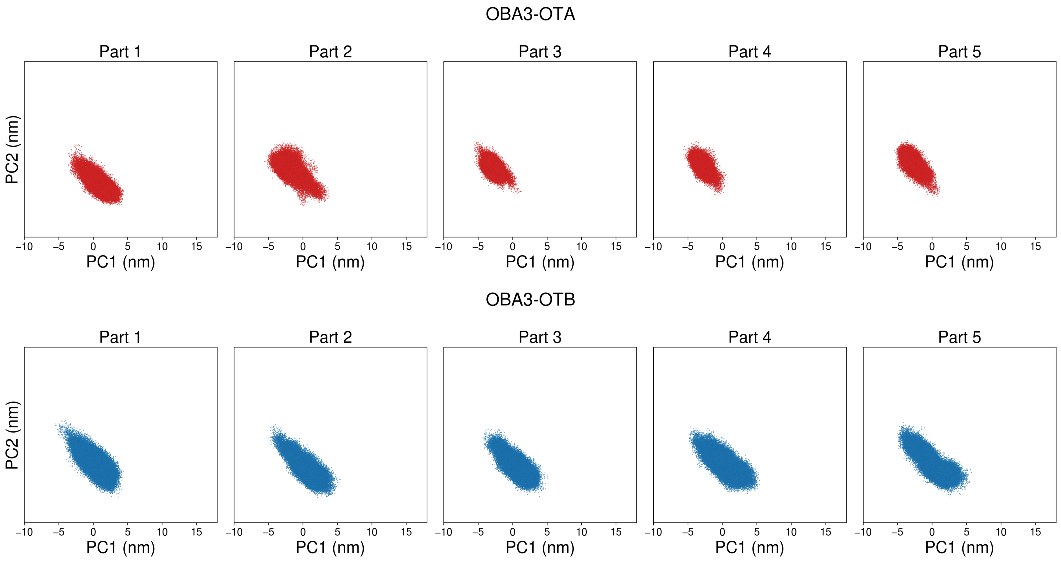

Figure S4: Projections of five consecutive blocks of the MD trajectories of OBA3-OTA and OBA3-OTB on the two-dimensional subspace described by the two first eigenvectors obtained by the PCA.

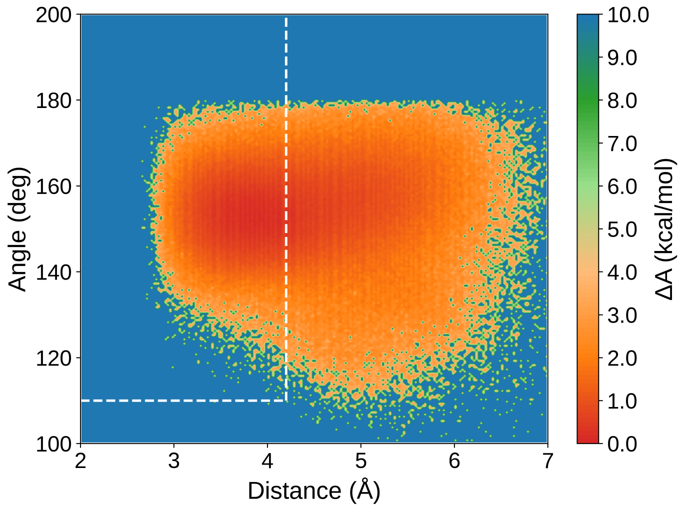

Figure S5: Combined distribution function for Cl-OP2 distance and C-Cl-OP2 angle to evaluate halogen interactions. Dotted lines show the threshold values of distance and angle.

Alchemical Free Energy

Table S4: Free energy (kcal mol^{-1}) difference obtained for each replica. $\Delta\Delta A$: relative free energy of binding for OBA3 using different integrators.

	$\Delta\Delta A$		
Rep.	CGI	BAR	JE
I	2.1	2.0	2.2
II	2.7	2.9	3.0
III	2.2	2.7	3.5
IV	3.7	3.5	4.0
V	3.7	3.7	3.4
mean	2.9 ± 0.7	2.9 ± 0.5	3.2 ± 0.6

Table S5: Free energy (kcal mol^{-1}) difference obtained for each replica. $\Delta\Delta A$: relative free energy of binding for OBA3 using different integrators. Simulation time of 50 ps and λ parameter adjustment rate 4×10^{-5} per time step. Results were obtained using 200 snapshots from the 20 ns equilibrium trajectory.

	$\Delta\Delta A$		
Rep.	CGI	BAR	JE
I	3.2	2.9	2.6
II	2.6	2.8	3.2
III	3.2	3.3	3.1
IV	3.4	3.3	4.3
mean	3.1 ± 0.3	3.1 ± 0.2	3.1 ± 0.3

Table S6: Free energy (kcal mol^{-1}) difference obtained for each replica. $\Delta\Delta A$: relative free energy of binding for OBA3 using different integrators. Simulation time of 500 ps and λ parameter adjustment rate 0.4×10^{-5} per time step. Results were obtained using 50 snapshots from the 20 ns equilibrium trajectory.

	$\Delta\Delta A$		
Rep.	CGI	BAR	JE
I	4.0	3.4	2.9
II	1.7	2.5	2.4
III	2.9	3.2	3.1
IV	3.7	3.3	3.7
mean	3.1 ± 0.9	3.1 ± 0.3	3.0 ± 0.5

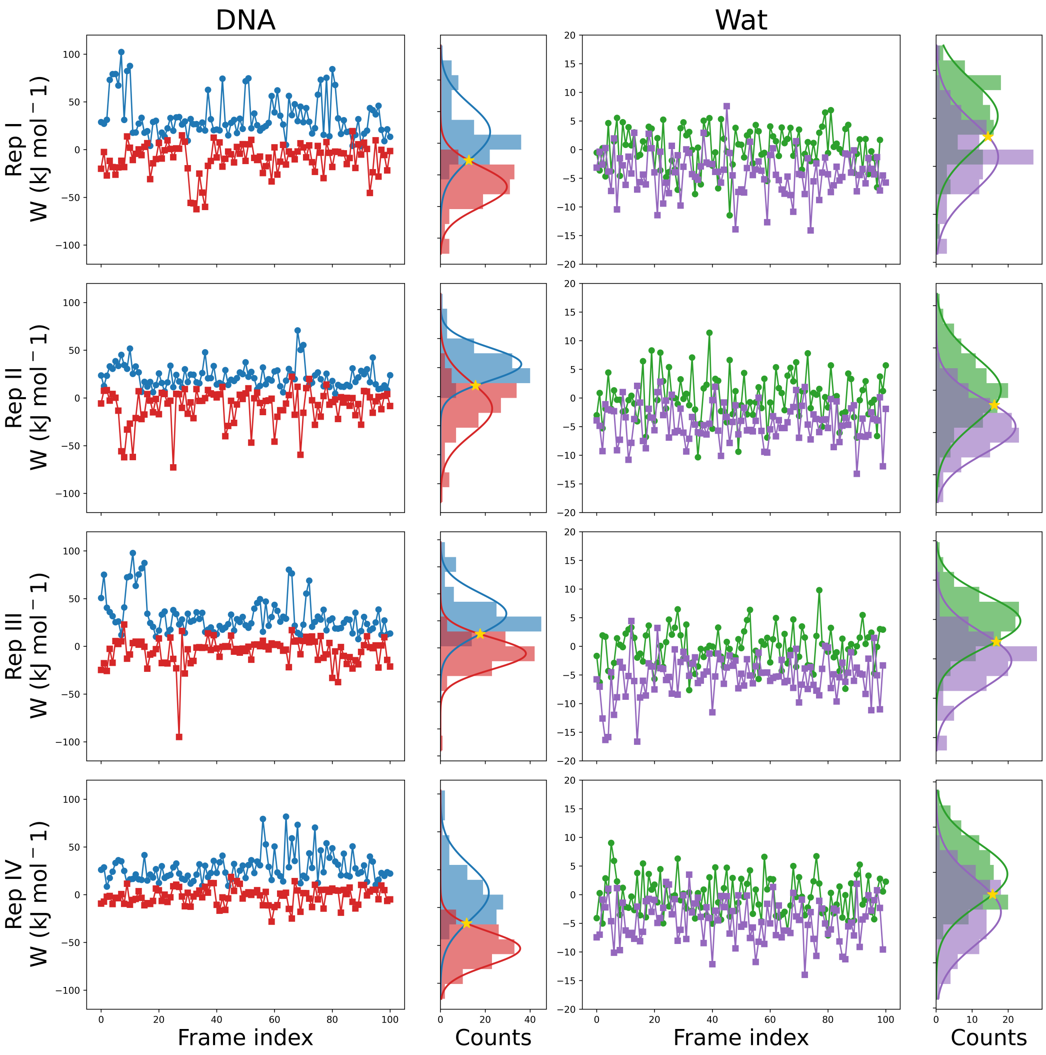

Figure S6: Calculated work values (W) for each extracted frame (100) in the alchemical free energy calculations of each replica, shown in OBA3 (left) and in water (right) systems (see Table 1). Forward (blue/green circles) and backward (red/purple squares) work distributions are plotted along with their respective histograms. The BAR integrator was used to estimate the free energy differences, indicated by the star on the histogram overlap.

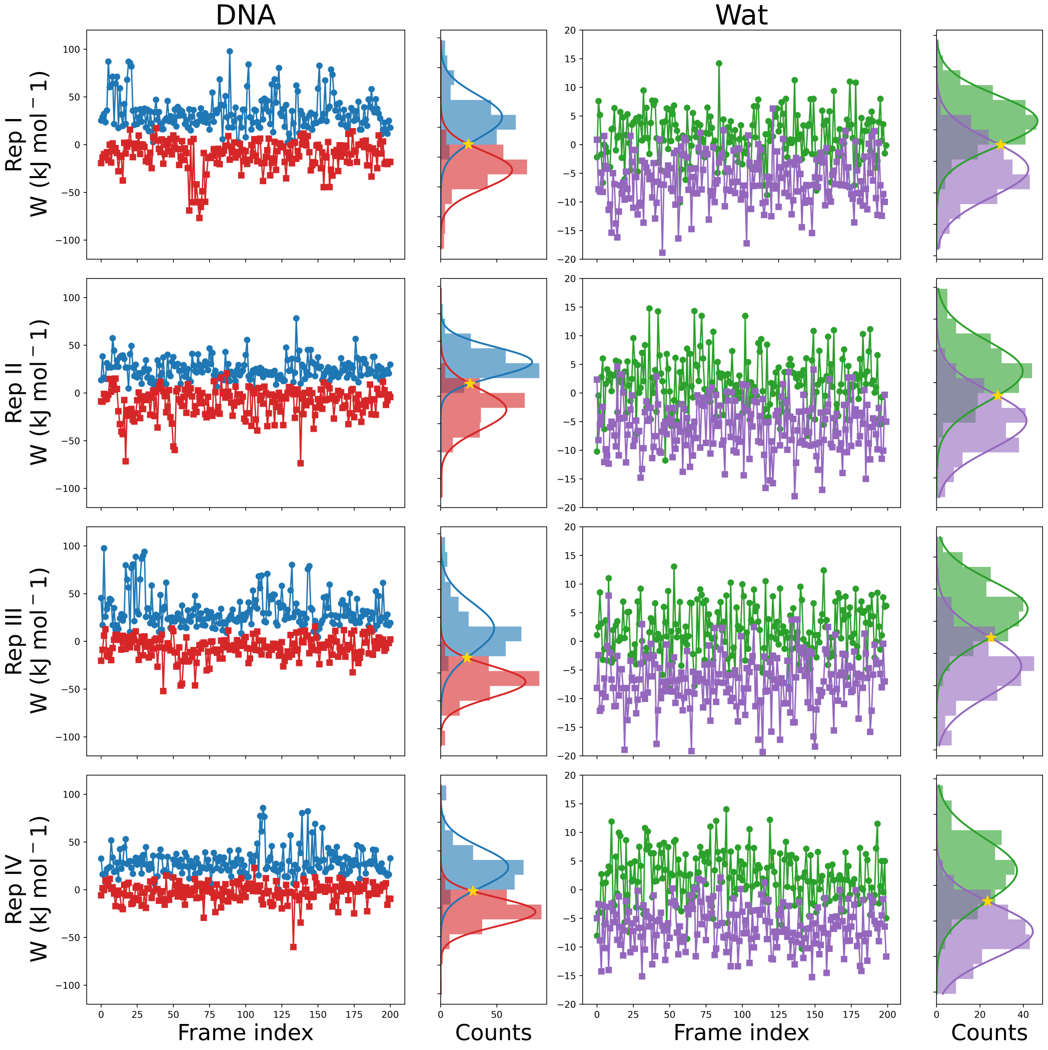

Figure S7: Calculated work values (W) for each extracted frame (200) in the alchemical free energy calculations of each replica, shown in OBA3 (left) and in water (right) systems (see Table S5). Forward (blue/green circles) and backward (red/purple squares) work distributions are plotted along with their respective histograms. The BAR integrator was used to estimate the free energy differences, indicated by the star on the histogram overlap.

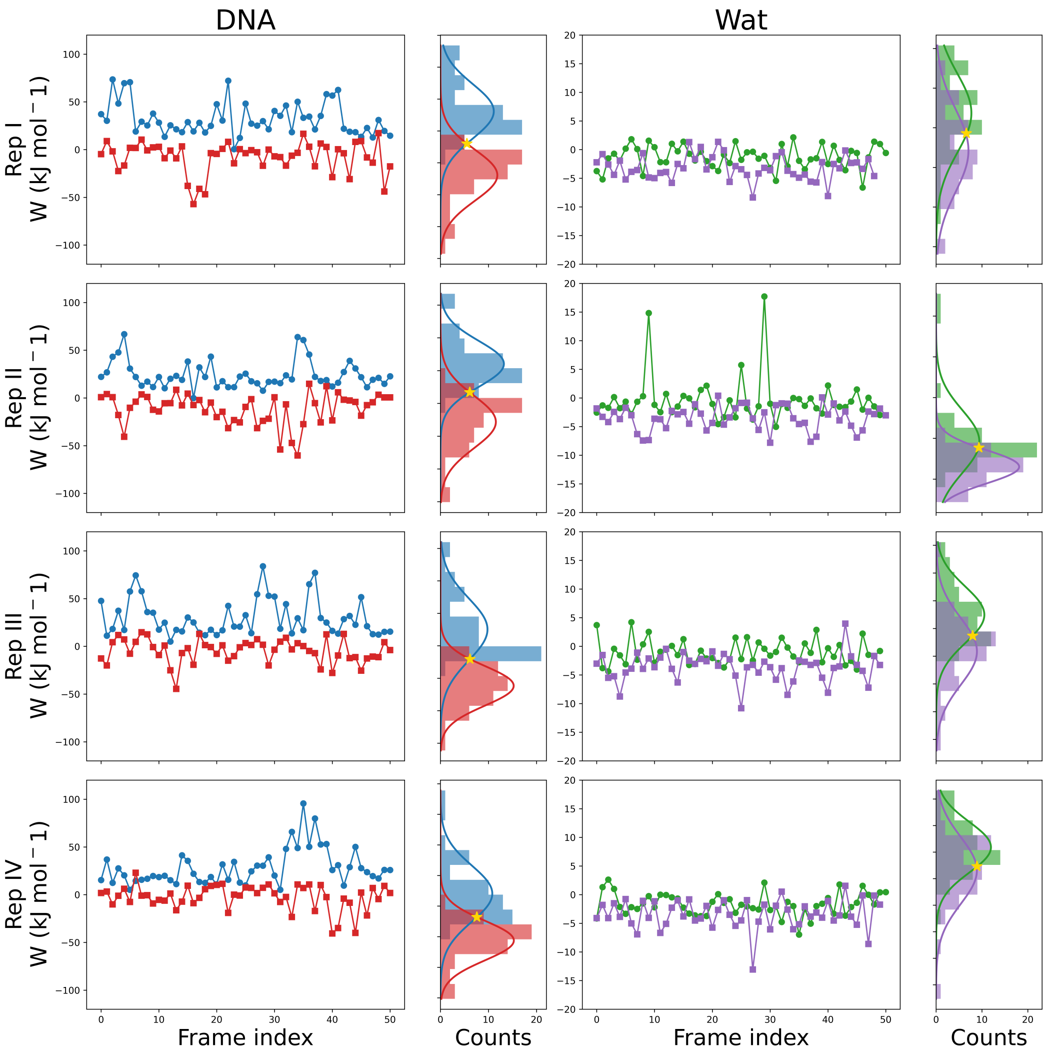

Figure S8: Calculated work values (W) for each extracted frame (50) in the alchemical free energy calculations of each replica, shown in OBA3 (left) and in water (right) systems (see Table S6). Forward (blue/green circles) and backward (red/purple squares) work distributions are plotted along with their respective histograms. The BAR integrator was used to estimate the free energy differences, indicated by the star on the histogram overlap.

OBA33

Structural Analysis

Table S7: Definition of the hydrogen bonds used for the OBA33 hydrogen bond analysis described in Figure 8.

Donor		Hydrogen		Acceptor	
Base	Atom	Base	Atom	Base	Atom
DC1	N4	DC1	H41	DG33	O6
DG2	N1	DG2	H1	DC32	N3
DG2	N2	DG2	H21	DC32	O2
DA3	N6	DA3	H61	DT31	O4
DT4	N3	DT4	H3	DA30	N6
DT4	N3	DT4	H3	DA30	N1
DG6	N1	DG6	H1	DT15	O4
DG7	N1	DG7	H1	DG26	O6
DG7	N2	DG7	H21	DG26	N7
DG8	N1	DG8	H1	DG12	O6
DG8	N2	DG8	H21	DG12	N7
DG10	N1	DG10	H1	DG24	N7
DG10	N2	DG10	H21	DG24	O6
DG12	N1	DG12	H1	DG17	O6
DG12	N2	DG12	H21	DG17	N7
DG13	N1	DG13	H1	DG7	O6
DG13	N2	DG13	H21	DG7	N7
DT15	N3	DT15	H3	DG6	O6
DG16	N1	DG16	H1	DG13	O6
DG16	N2	DG16	H21	DG13	N7
DG17	N1	DG17	H1	DG25	O6
DG17	N2	DG17	H21	DG25	N7
DC18	N4	DC18	H41	DG24	O6
DG24	N1	DG24	H1	DC18	N3
DG24	N2	DG24	H21	DC18	O2
DG25	N1	DG25	H1	DG8	O6
DG25	N2	DG25	H21	DG8	N7
DG26	N1	DG26	H1	DG16	O6
DG26	N2	DG26	H21	DG16	N7
DA30	N6	DA30	H61	DT4	O4
DT31	N3	DT31	H3	DA3	N1
DC32	N4	DC32	H41	DG2	O6
DG33	N1	DG33	H1	DC1	N3
DG33	N2	DG33	H21	DC1	O2

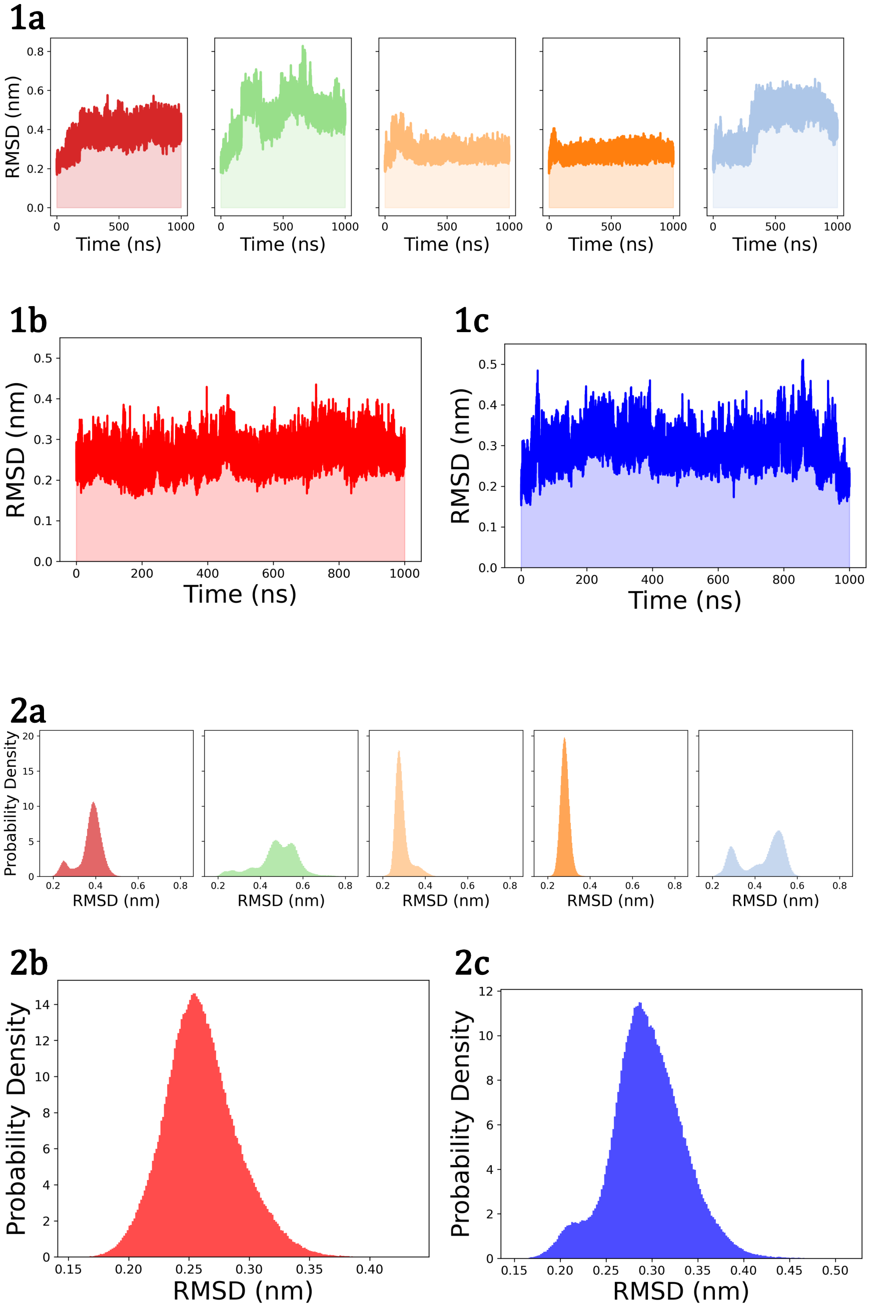

Figure S9: Root Mean Square Deviations computed on the phosphorous atoms of the OBA33 aptamer, with respect to the centroid of the models in PDB (7W9N). Both evolution in time and distributions ar shown. Panels: 1a) ligand-free OBA33 (5 replicas), 1b) OBA33-OTA complex, 1c) OBA33-OTB complex, 2a) distribution of ligand-free OBA33 RMSD (5 replicas), 2b) distribution of OBA33-OTA complex RMSD, 2c) distribution of OBA33-OTB complex RMSD.

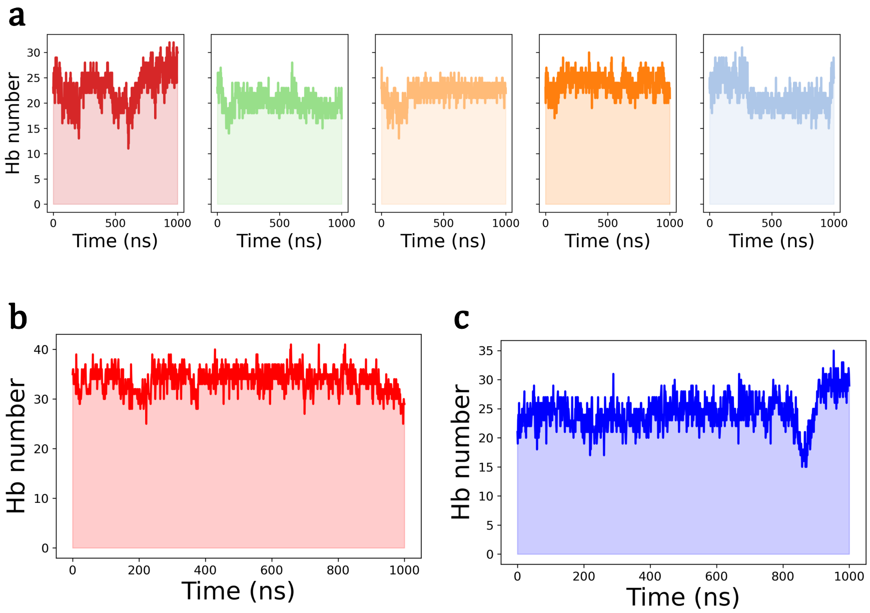

Figure S10: Hydrogen bonds sampled among nucleobases of the OBA33 aptamer throughout the simulation for (a) the 5 replicas of the ligand-free OBA33, (b) the OBA33-OTA complex, (c) the OBA33-OTB complex.

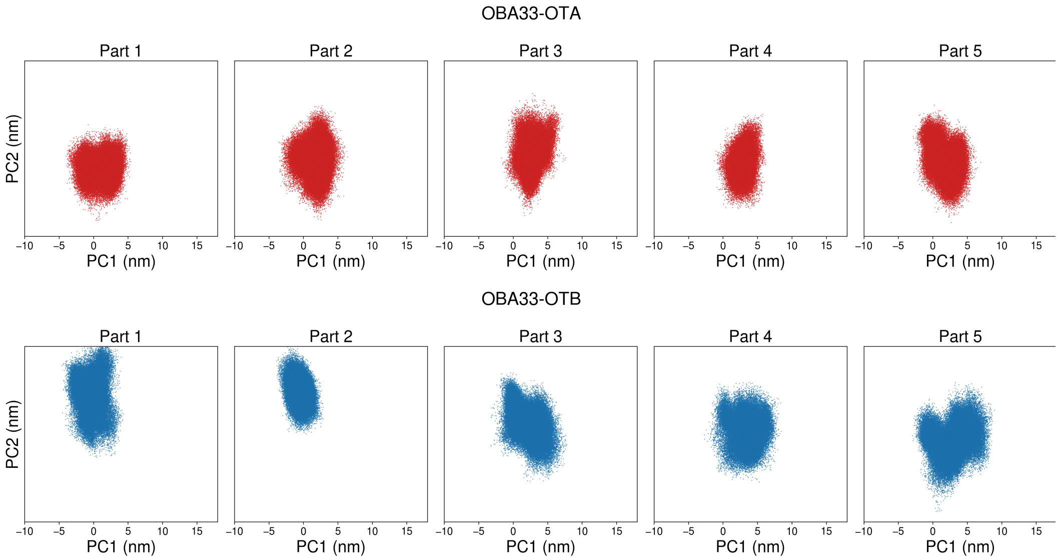

Figure S11: Projections of five consecutive blocks of the MD trajectories of OBA33-OTA and OBA33-OTB on the two-dimensional subspace described by the two first eigenvectors obtained by the PCA.

Alchemical Free Energy

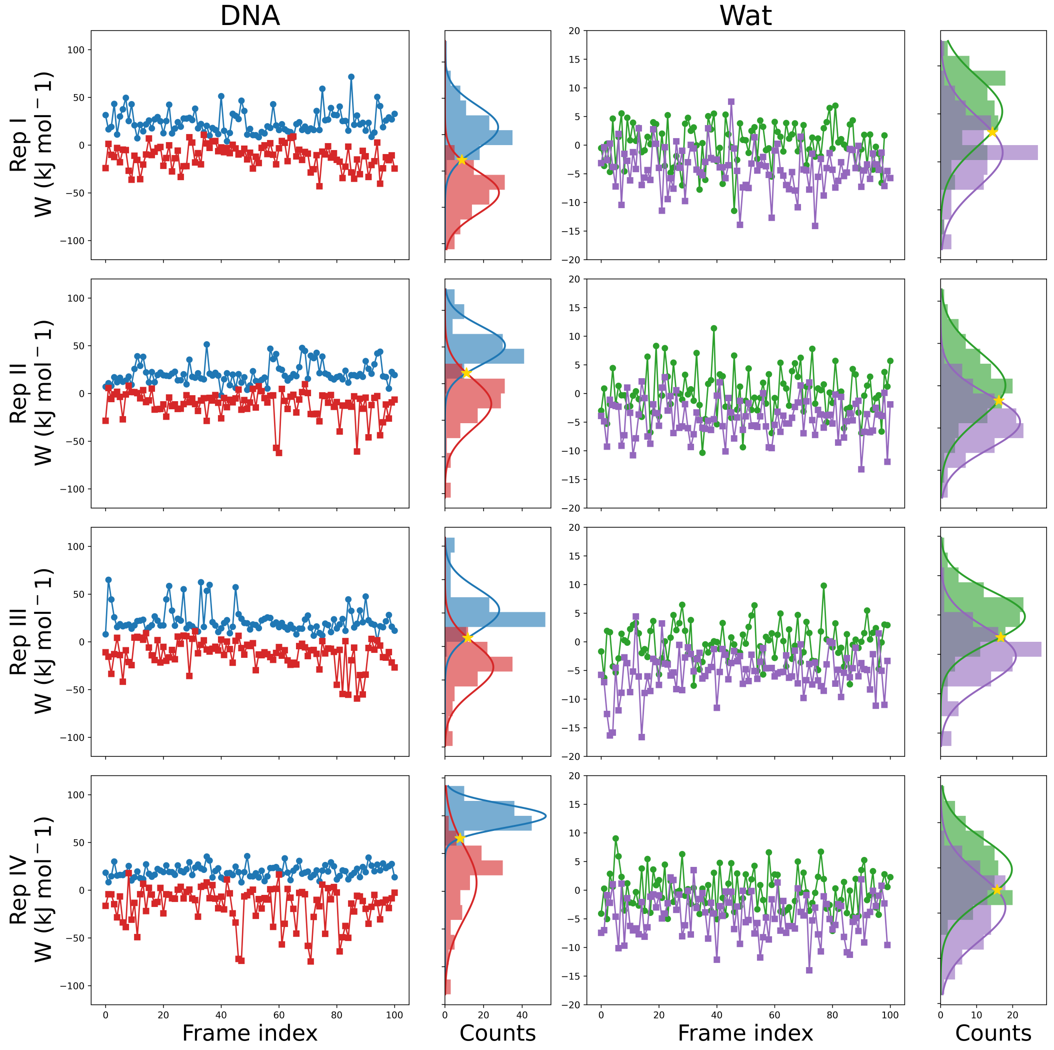

Figure S12: Calculated work values (W) for each extracted frame in the alchemical free energy calculations of each replica, shown in OBA33 (left) and in water (right) systems (see Table 2). Forward (blue/green circles) and backward (red/purple squares) work distributions are plotted along with their respective histograms. The BAR integrator was used to estimate the free energy differences, indicated by the star on the histogram overlap.

Table S8: Free energy (kcal mol^{-1}) difference obtained for each replica. $\Delta\Delta A$: relative free energy of binding for OBA33 using different integrators.

	$\Delta\Delta A$		
Rep.	CGI	BAR	JE
I	2.0	2.2	2.3
II	1.5	1.9	1.7
III	1.6	2.2	2.2
IV	2.6	2.6	3.3
V	1.8	1.9	1.8
mean	1.9 ± 0.4	2.2 ± 0.3	2.3 ± 0.6

References

(1) Ibrahim, M. A. Molecular mechanical study of halogen bonding in drug discovery. *J. Comput. Chem.* **2011**, *32*, 2564–2574.
